# A lignan compound regulates LPS modifications via PmrA/B signaling cascades to potentiate colistin efficacy *in vivo*

**DOI:** 10.1371/journal.ppat.1013843

**Published:** 2025-12-29

**Authors:** Qiuyue Diao, Zixing Zhong, Qin Zhong, Yidan Cao, Xiaona Fan, Yujiao Liang, Huihui Zhang, Zehua Cui, Xinlei Lian, Xiaoping Liao, Donghao Zhao, Jian Sun, Hao Ren

**Affiliations:** 1 State Key Laboratory of Animal Disease Control and Prevention, College of Veterinary Medicine, South China Agricultural University, Guangzhou, China; 2 Guangdong Provincial Key Laboratory of Veterinary Pharmaceutics Development and Safety Evaluation, South China Agricultural University, Guangzhou, China; 3 Guangdong Provincial Key Laboratory of Veterinary Pharmaceutics, Development and Safety Evaluation, South China Agricultural University, Guangzhou, China; VIT University: Vellore Institute of Technology, INDIA

## Abstract

There has been a substantial gap between drying antibiotic pipeline and ongoing antibiotic resistance crisis, necessitating approaches to revitalize existing antimicrobials to meet unmet clinical demand for viable treatments. Herein, a lignan compound, magnolol, was identified that profoundly potentiates colistin (CS) to eradicate Gram negative bacteria and curb the development of resistance under host-mimicking condition. The mechanistic study showed that magnolol is able to disrupt PmrA/B two component signaling by dissociating the PmrA regulator protein from its cognate DNA including *eptA* and *arnT*. This action blocks the PmrA/B-dependent protective modifications of lipopolysaccharide (LPS) to reduce the net charges of bacterial membrane, thereaby facilitating its electrostatic interaction with CS. MAG-facilitated enhancement of CS binding promotes the formation of toroidal pores in the bacterial membrane, which in turn triggers rapid bacterial death by inducing lethal cytoplasmic contents leakage. In sum, this work not only illustrates the great potential of untapped phytoconstitutes such as magnolol in confronting antibiotic resistance but also reveals that silencing PmrA/B signaling as a favorable strategy to potentiate CS activity *in vivo*.

## Author summary

As one of the last-line antibiotics, the colistin holds clinically indispensable for treating against MDR Gram negative pathogens but is limited by toxicity and growing resistance. Importantly, certain pathogens can develop colistin resistance in response to in-host stimuli, thereby offsetting colistin efficacy *in vivo*. In this context, well-designed adjuvants that potentiate its activity *in vivo* allow colistin to be used at lower, safer and more precise doses, and help extend the lifespan of this last-line therapy. Herein, we applied a high throughput screening based on a host-mimicking LPM medium and identified a lignan compound, magnolol (MAG), that is able to potentiate colistin efficacy *in vitro* and *in vivo*. It was found that this potentiation relies on MAG’s ability to paralyze PmrA/B-dependent membrane modifications to facilitate the colistin binding and destabilizing bacterial membrane. We also propose a new mode of PmrA/B inactivation for MAG, in which it acts as a molecular decoy to dissociate the PmrA regulator protein from its target DNA. These findings suggest that the phytochemicals, exemplified by the MAG, are untapped source for potential antibiotic adjuvants and trapping the transcriptional regulation of the PmrA/B two component system may present a new mechanistic paradigm for developing next-generation colistin adjuvants.

## Introduction

Antibiotics have become one of most important medications allies in the fight against pathogen infections. Unfortunately, the unguided use of antibiotics in clinics and agriculture together lead to the growing problem of antimicrobial resistance (AMR), which has become an increasingly global threat in recent decades [1]. Replenishing the antibiotic pipeline has emerged as a top priority to tackle AMR, and the discovery of darobactin, teixobactin, halicin, clovibactin and other new antibiotics are milestone efforts to curb the full-blown post-antibiotic era [2–5]. Hitherto, most of these lead molecules are undergoing the preclinical trials and only a handful of them such as cefiderocol have been licensed. In light of the void in antibiotic pipeline, several innovative approaches have emerged to address the urgent need for viable therapies. Such strategies, including antivirulence agents, decolonization approaches, host-directed therapies, are believed to alleviate the bacterial infection under relatively low selection pressure and open new perspectives to counteract the AMR crisis to some extents [6–8]. However, these approaches each have advantages and limitations, the ideal solutions are expected to balance efficacy, cost, safety and ease-of-handling.

In this regard, attempts to revitalize existing antibiotics are sought to offer a promising opportunity to combat antibiotic resistance with merits in eco-friendliness and well-established safety profiles [9]. To maximally exploit the therapeutic potential of existing drugs, the synergistic combinations are of most promise and feasibility [10]. In the efforts to expand the arsenal of combination therapy, multiple compounds have been identified as effective adjuvants that potentiate antibiotic activity. For example, β-lactamase inhibitors, such as clavulanate and avibactam, have a good history to restore the β-lactam potency against drug-resistant pathogens that do not respond to single treatment [11]. In addition, membrane active agents as reported such as SLAP-25 have also been reported to potentiate antibiotic killing by facilitating the uptake of structurally-diverse antibiotics including tetracycline, quinolone and cephalosporin classes [12]. Recently, bacterial responses to specific conditions have also been shown to manipulate the antibiotic susceptibility in many species. A good example is our previous investigation, in which the aminoglycoside-induced protein misfolding promoted the prodrug activation and efficacy of nitrofurantoin via CpxA/R-MarA/SoxS-NsfA/B regulatory cascade [13]. Although there have been many successes in combinatorial antibiotic therapies, it still should be noted that most established drug pairs, especially the early attempts, were assembled an in *ad hoc* manner without rigorous genetic explanation [14]. Therefore, genetically determined drug synergies are particularly attractive from the therapeutic standpoint, as they may reduce the potential for unintended outcomes.

Among all antibiotics in use, colistin (CS) holds extensive clinical importance to tackle multidrug-resistant Gram-negative bacteria due to its unique mode of action that bypasses most traditional resistance mechanisms However, the clinical utility of CS has been increasingly challenged by the acquired resistance mediated by chromosomal mutations in *mgrB* or by transferable *mcr* variants. Additionally, certain bacterial species exhibit adaptively inducible resistance against CS killing by modifying the lipid A moiety of lipopolysaccharide (LPS) in response to environmental stimuli [15]. These factors collectively contribute to the suboptimal clinical responses of CS treatment, with up to 70% of patients experiencing recurrent infection during or after CS therapy [16–18]. Hence, innovations in CS-based treatment paradigm is urgently needed. To address this demand, a growing number of CS adjuvants have been screened. In these pioneering works, the ionophore PBT-2, the gold drug auranofin, and others were found to potentiate CS activity by targeting either acquired or intrinsic resistance mechanisms [19,20]. Of note, those inhibit LPS-modification mechanisms are particularly valuable because they target the proximate biochemical changes that directly prevent CS binding. For instance, Barker et al. repurposed several eukaryotic kinase inhibitors as CS adjuvants by reducing cationic substitutions on LPS, enhancing CS activity against both CS-sensitive and –resistant isolates [21]. These efforts clearly demonstrate the promise of combining CS with its potentiators in improving the pathogen clearance and clinical outcomes. Nonetheless, most current potentiators were identified by standard antimicrobial susceptibility testing (AST) using universal rich media, which poorly replicate the *in vivo* conditions. This notion indicates that the commonly-used screening procedures for CS adjuvants should be revisited, to maximally avoid inadvertent exclusion of compounds with genuine *in vivo* potency.

To better bridge the gap between rising demand and limited number of viable treatment options, herein we reported the discovery of a lignan compound, magnolol (MAG) as a potent CS adjuvant especially under host-mimicking conditions. Further study revealed that MAG was able to pause the PmrA/B-dependent LPS modification through dissociating the PmrA regulator from its target genes including *eptA* and *arnT*. Consequently, the MAG-suppressed LPS modification enhances CS binding and promotes destabilization of bacterial membrane, ultimately killing pathogens by enhanced toroidal pore formation. Together, this study provides CS-MAG combination as a viable therapeutic regimen against Gram negative bacteria and sheds light on the new mechanistic paradigm to potentiate CS by manipulating PmrA/B signaling via allosteric control.

## Results

### Chemical screening identified MAG as a viable adjuvant to CS under host condition

**Fig 1:**
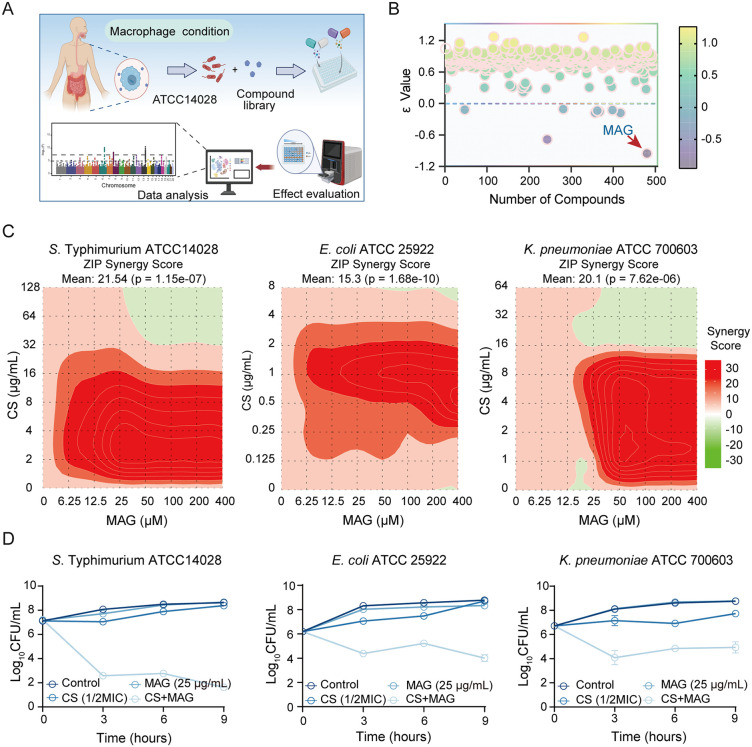
Chemical screening identified MAG as a viable CS adjuvant under host condition. **(A)** Scheme of the primary screening protocol based on host-mimicking condition (This illustration was created in Biorender. H.R, https://BioRender.com/sbsz5e9). **(B)** MAG was found as the most potent adjuvant to CS activity (50 µM of each chemical for selection including MAG, ε̃ value, which calculated based on the relative growth of bacterium under mono- or combination therapy, was used to illustrate the interaction between CS and screened chemicals). **(C)** The synergy landscape of MAG with CS on three model strains (*S.* Typhimurium ATCC14028, *E. coli* ATCC25922, *K. pneumoniae* ATCC700603), calculated by the open-source drug interaction prediction toolkit: Synergyfinder 3.0. **(D)** MAG potentiated the bactericidal activity of CS on CS-resistant Gram-negative bacteria (Selected strains from late exponential phases were treated by CS at 1/2 MIC alone or with MAG at 25 µg/mL, results presented as mean ± s.d of data from 3 biologically independent experiments).

In general, agents that potentiate CS under conditions mimicking natural infections are more likely to translate into effective therapeutic. Therefore, the low-phosphate, low-magnesium medium (LPM) was applied to select CS adjuvants that expected effective to be *in vivo*. In this host-mimicking medium, the indicator strain ATCC 14028s (*Salmonella* Typhimurium, CS-sensitive) was readily 32-fold more resistant to CS compared with its susceptibility in rich MH medium (Table 1). To rapidly yield candidates of interest in rapid and high-throughput manner, a previously published protocol [22] was applied an in-house screening of chemicals from lab collection according (Fig 1A). In this protocol, potentiation was indicated by the ε̃ value which was calculated by based on the normalized bacterial growth expose to single drug or drug combination (detailed computational procedure shown in Methods section). With a set cut-off of -0.5, 2 hits were identified from the primary screening, among which MAG was found to be most potent (Fig 1B). The MAG is a herbal constituent of the bark of Magnolia officinalis and possess a typical lignan skeleton (S1 Fig). Although this molecule is known for its multifunctional roles in modulating anti-inflammatory, anti-oxidative and anti-cancer activities in eukaryotic cells, its potential to potentiate antibiotics remains largely unexplored. In this regard, the potential interaction between MAG and CS was further validated using SynergyFinder [23], which quantitatively assessed interaction across three model bacteria targeted by CS (*S. Typhimurium* ATCC 14028; *K. pneumoniae* ATCC 700603; *E. coli* ATCC 25922). By analyzing the full dose–response matrices of MAG–CS combinations, SynergyFinder generated two-dimensional synergy landscapes, allowing direct comparison of interaction patterns across strains. The results indicated that a profound interaction between MAG and CS was elucidated on selected strains with the average synergy scores (as indicated by the Zero Interaction Potency model, ZIP) ranging from 15.3 to 21.54 (Fig 1C). This potentiation on colistin by MAG was re-confirmed by the classic checkerboard assay as the fractional inhibitory concentration index (FICI) were observed from 0.125 to 0.281 (S2 and S3 Figs). It was noted that the potentiation of MAG only observed in the LPM. Its suboptimal activity in the rich medium suggested that it selectively tackled the bacterial intrinsic resistance mechanism in response to host-mimicking condition (S4 Fig). Furthermore, a directly enhanced bactericidal action supports thse findings. To this end, time-killing assays were performed on aforementioned model strains treated with CS at sublethal dose (1/2 MIC, 16 µg/mL) with or without MAG (25 µg/mL). As expected, the model strains were not indeed affected by the sublethal CS alone, yet were rapidly killed by the MAG-CS combination comparing with the monotherapy (Fig 1D). Collectively, the screening identified the MAG as a potential lead molecule to potentiate CS activity under host condition.

**Table 1:** Bacterial strains used in the current study.

Organism	Strain	Medium	MIC	
			MAG (mM)	CS (μg/mL)
*Salmonella* Typhimurium	ATCC14028	MH	>1	1
		LPM	>1	32
*Escherichia coli*	ATCC25922	MH	1	2
		LPM	1	4
*Klebsiella pneumoniae*	ATCC700603	MH	1	2
		LPM	1	32
*Salmonella* Typhimurium	ATCC14028 Δ*pmrA*	MH	>1	0.5
		LPM	>1	1
*Salmonella* Typhimurium	ATCC14028 Δ*pmrB*	MH	>1	0.5
		LPM	>1	1
*Salmonella* Typhimurium	ATCC14028 Δ*eptA*	MH	>1	0.5
		LPM	>1	1
*Salmonella* Typhimurium	ATCC14028 Δ*arnT*	MH	>1	0.5
		LPM	>1	0.5
*Salmonella* Typhimurium	ATCC14028 Δ*phoP*	MH	1	0.5
		LPM	0.2	2
*Salmonella* Typhimurium	ATCC14028 Δ*phoQ*	MH	1	0.5
		LPM	0.2	2

### MAG restores CS activity against resistant bacteria and impedes resistance development

**Fig 2:**
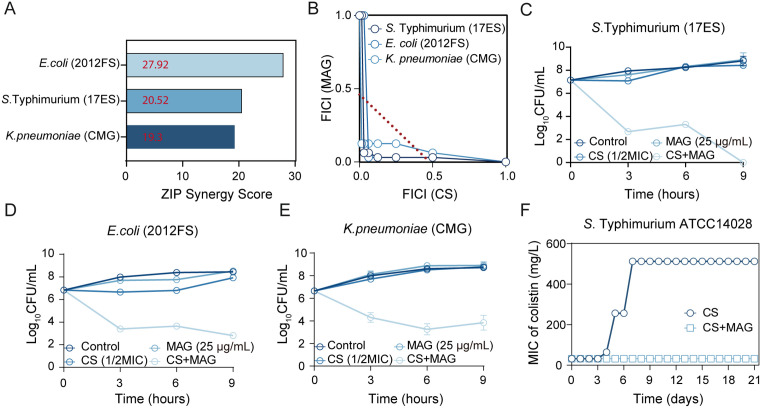
MAG restores CS activity against various CS-resistant isolates and curb the resistance establishment. **(A)** The ZIP synergy score of MAG-CS combination on three CS-resistant isolates. **(B)** The isobolograms of the combination of CS and MAG against different colistin-resistant isolates; MAG potentiated the bactericidal activity of CS (1/2 MIC) on the resistant *S.* Typhimurium **(C)**, *E. coli* **(D)**, *K. pneumoniae* **(E)**. Results from the killing assay were presented as mean ± s.d of data from 3 biologically independent experiments. **(F)** Presence of MAG (25 µg/mL) curbed resistance development in bacteria upon CS exposure (1/4 MIC, 8 µg/mL).

Given that the resistance to CS has been wide-spread among the Enterobacteriaceae in both clinic settings and livestock, it is of significance to explore its activity in CS-resistant strains. Generally, acquired colistin resistance mediated by plasmid-borne *mcr* genes or chromosomal mutations enables bacteria to covalently modify LPS thereby reduce CS binding [24]. To illustrate the potency of MAG against colistin-resistant isolates, strains bearing *mcr* gene (*mcr*-positive *S.* Typhimurium 17ES and *E. coli* 2012FS) or chromosomal *mgrB* mutation (*K. pneumoniae* CMG) were tested. The results indicated that the MAG was able to enhance CS activity against these CS-resistant isolates as shown by desirable ZIP score ranging from 19.30 to 27.92 (Figs 2A and S5). Consistent with this, the isobologram analysis confirmed that synergy was across different strains and the more pronounced in CS-resistant bacteria than in CS-sensitive strains, with FICI ranging from 0.09375 to 0.15625. (Figs 2B and S6). To further characterize the interaction between MAG and CS on the CS-resistant strains, the time-killing assays were performed. In these CS–resistant strains, CS monotherapy had negligible impact on bacterial viability over time yet the CS-MAG combination promptly killed the tested strains with 2–4 additional orders of magnitude within 9 h (Fig 2C–2E). These findings strongly indicate that MAG can also potentiate the bactericidal activity of CS against these strains. An optimal treatment is also expected to minimize the evolution of resistance under selection pressure. Thus, the bacteria were challenged by the CS (1/4 MIC, 8 µg/mL) in the serial passage with or without MAG. The CS resistance was emerged soon after 5^th^ passage and the MIC value exceeding 512 mg/L after 8^th^ generation (Fig 2F). In contrast, the combination with MAG significantly reduced possibility for resistance development as no increase in MIC was observed throughout the experiment. The whole-genome sequencing revealed that the bacteria exposed to CS passage alone rapidly enriched with mutations that likely confer CS resistances, while the evolution in CS-MAG treated bacteria was greatly suppressed (S7 Fig). Taken together, these results indicate that the MAG holds special potential as an adjuvant to restore CS activity in resistant bacteria and curbs the resistance development under host-mimicking conditions, underscoring the importance of elucidating its mode of action.

### Enhanced toroidal pore formation dictates the potentiation of MAG on CS

**Fig 3:**
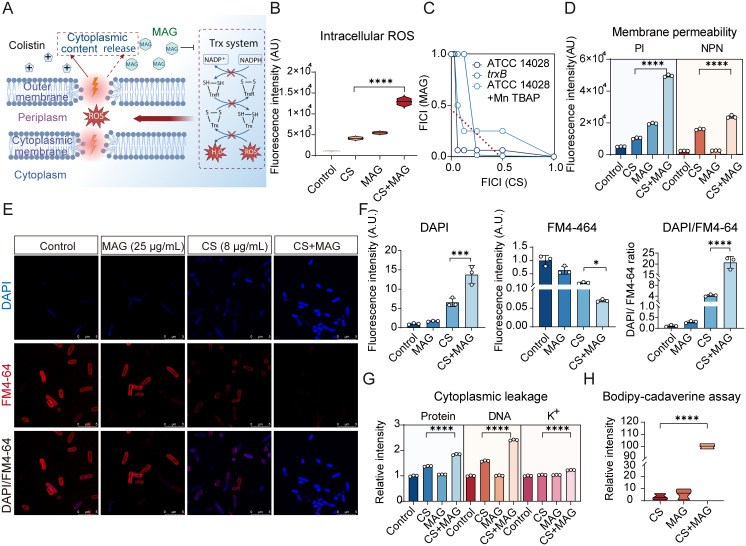
Enhanced toroidal pore formation dictates the potentiation of MAG on CS. **(A)** Schematic illustration of mode of action of CS (This illustration was created in Biorender. H.R, https://BioRender.com/zw4qvd3). **(B)** ROS measurements in bacterial cells treated by CS, MAG and their combinations. **(C)** MAG attacked *trx* system to disrupt bacterial redox balance only partially responsible for MAG-CS synergy. **(D)** MAG enhanced CS-mediated membrane permeability alteration (PI: Propidium Iodide, indicator probe for inner membrane permeability; NPN: N-phenyl-1-naphthylamine, indicator probe for outer membrane permeability). **(E)** Microscopy-based bacterial cytological profiling on cells treated by CS, MAG and their combination: FM4-64 (red cell membrane stain), DAPI (blue DNA stain). **(F)** Quantitative analysis of bacterial cytological profiling unveiled increased toroidal pore formation in bacterial cells treated by CS + MAG. **(G)** MAG-assisted toroidal pore formation facilitated cytoplasmic contents leakage. **(H)** BODIPY-tagged cadaverine displacement assay revealed MAG enhanced CS binding on bacterial membrane. All assays performed in B, D, F, G, H were in three biologically independent experiments, and the mean ± s.d. is shown, n = 3. *p < 0.05, **p < 0.01, ***p < 0.001, ****p < 0.0001, determined by nonparametric one-way ANOVA analysis.

With colistin potentiation activity of MAG confirmed, it is of essential interest to elucidate the underlying mechanism. It has been well documented that CS elicits bactericidal action by promoting toroidal pore formation and subsequent reactive oxygen species (ROS) generation (Fig 3A). Since MAG was reportedly to perturb the cellular redox balance by inhibiting thioredoxin reductase (*Trx* system) in many bacterial species, we first postulated that MAG might potentiate CS activity by targeting *Trx* to augment ROS production. Using a ROS-sensitive dye, it was shown that combination of MAG and CS substantially elevated the ROS production compared to CS or MAG monotherapy (Fig 3B). This promoted us to further examine whether the *Trx* insults and latter ROS generation dictated the interaction between CS and MAG. To this end, a checkerboard assay was performed on *trxB*-deficient mutant. Nonetheless, MAG was still found to potentiate CS, albeit the potentiation was slightly dampened with increased FICI (0.265625). The interaction between MAG and CS was also probed in the inoculum where MnTBAP was supplemented to scavenge the excessive ROS. In concert with the prior result, quenching ROS in treated bacterial cell was not able to fully revert the potentiation activity of MAG (Figs 3C and S8). These results together suggested that additional mechanism beyond ROS augmentation via *Trx* inhibition contribute to MAG-mediated potentiation. In this regard, we then hypothesized that the MAG might promote the toroidal pore formation, a process equally important for CS-mediated killing. Membrane-permeability dyes revealed that the combination with MAG significantly increased both outer and inner membrane permeability, indicative of enhanced toroidal-pore induction and lipid bilayer destabilization (Fig 3D). To better contextualize MAG-assisted pore forming action, fluorescence microscopy-based bacterial cytological profiling (BCP) was performed on bacterial cells treated by CS, MAG or their combination. Dual staining with the cell membranes and nucleoids using FM4–64 and DAPI, CS plus MAG sharply reduced the red membrane fluorescence yet increase the blue cytoplasmic fluorescence (Fig 3E). As quantified in Fig 3F, the altered florescence ratio indicated that MAG enhances CS to inevitably destroy the membrane integrity and synchronously facilitates entry of extracellular substances. Because pore formation is often accompanied by leakage of cytoplasmic contents leakage, an important determinant for bacterial cell death. Hence, we next probed the leakage of cytoplasmic contents including proteins, DNA and ions (potassium as proof-of-concept) in the bacterial cells exposed to CS alone or the MAG-CS combination. It was observed that a significant loss of these important cytoplasmic micro- and macromolecules following the addition of combination, whereas the CS alone triggered only modest leakage (Fig 3G). These findings echo BCP observation and strongly corroborated the conclusion that MAG potentiates CS by promoting membrane damage and subsequent toroidal pore formation. Finally, we sought to understand the rationale behind the enhanced pore formation and cytoplasmic leakage. As a membrane-acting agent, CS is known to bind directly to negatively charged LPS to form crystalline structures and toroidal pores segregated from zwitterionic phospholipids [25]. This led us to hypothesize that MAG may assist the membrane-binding actions of CS, thereby promoting pore formation. To test this, a previously-reported membrane binding assay using BODIPY-tagged cadaverine (BC) probe, which also binds to lipid A phosphate groups and fluoresces upon displacement by CS, was employed. As expected, incorporation of MAG markedly increased the capability of CS to bind membrane, as indicated by elevated fluorescence due to BC displacement (Fig 3H). Collectively, these results collectively highlight the primary mechanism of MAG-mediated CS potentiation in which MAG enhances CS binding to promote the toroidal pore formation and consequently leads to detrimental cytoplasmic content leakage.

### MAG obstructs LPS modifications to enhance electrostatic attraction of CS

**Fig 4:**
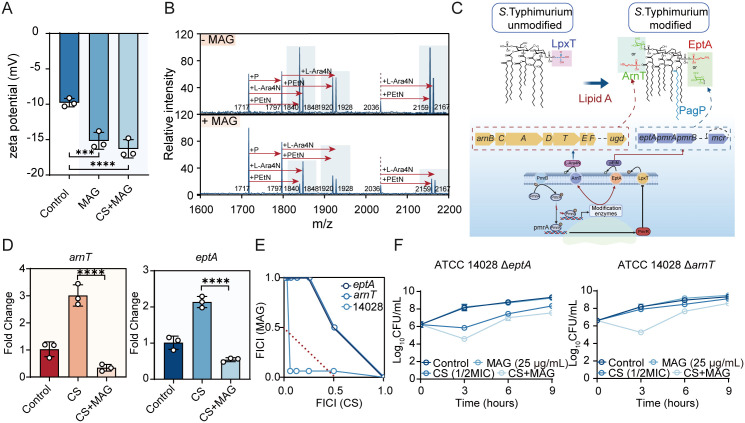
MAG blockades the LPS remodeling to enhance CS activity. **(A)** MAG treatment modulate bacterial membrane net charge to enhance electrostatic interaction with CS. **(B)** MALDI-TOF elucidated the bacterial membrane modifications in response to MAG treatment. **(C)** Scheme of protective LPS modification to offset the CS binding (This illustration was created in Biorender. H.R, https://BioRender.com/it3jbp5). **(D)** Transcriptions of genes responsible for bacterial membrane lipid modification were dampened by MAG. **(E)** Synergism between MAG and CS was diminished by deficiency of *eptA* and *arnT*. **(F)** MAG was not able to enhance bactericidal activity of CS in strains deficient in *eptA* and *arnT.* All assays performed in A, D and F were in three biologically independent experiments, and the mean ± s.d. is shown, n = 3. *p < 0.05, **p < 0.01, ***p < 0.001, ****p < 0.0001, determined by nonparametric one-way ANOVA analysis.

As shown in aforementioned section, the increased affinity of CS to bacterial membrane accounts for the enhanced CS activity mediated MAG. Our next task was therefore to decipher the determinant that responsible for this MAG-induced increase in CS binding. The mechanism of CS fundamentally relies on the interactions between its positively charged amino acid residues and the negatively charged lipid groups of LPS on the membrane [26,27]. Therefore, the net surface charge is essential for electrostatic interaction between CS and bacterial cells. To address this, the surface charge of bacterial cells treated with MAG were examined. As illustrated in Fig 4A, supplementation of MAG significantly reduced the surface charge of cell membrane, rationalizing the increased LPS binding. The precedent experiences established that bacterial surface charge is generally associated with LPS modifications [28]. Hence, the MALDI-TOF was then applied to explore the LPS profiles of bacteria with or without MAG treatment. Intriguingly, all m/z peaks corresponding to PEtN and L-Ara4N modifications were substantially reduced in the presence of MAG (Fig 4B). These chemical modifications provide the positive charges to the LPS and are conducted by *eptA*- and *arnT*-encoded enzymes in many Gram-negative bacteria to confer resistances against environmental stresses as well as cationic peptide killing, favoring bacterial survival under such conditions (Fig 4C). It was therefore plausible that MAG downregulates these regulations to impede cationic substitution of negative groups in LPS. To clarify this, transcriptions of genes responsible for LPS modifications were measured by RT-qPCR and transcriptional reporter assays. As can be seen in Figs 4D and S8, the expressions of target genes were induced by CS as part of defensive mechanism but markedly suppressed by MAG-CS combination. These results suggested that EptA/ArnT-directed LPS modifications acts as the central player in MAG-driven CS potentiation. To further validate the role of *eptA/arnT* duo, we further expanded the analysis of MAG-CS interaction in the mutants defective in *eptA* and *arnT*. As indicated by the isobolograms, deletion of these genes readily abolished the synergistic interaction between MAG and CS (Fig 4E). In line with the checkerboard assay, the time-killing assay confirmed that loss-of-functions in LPS modification diminished the MAG-mediated potentiation (Fig 4F). Taken together, these results suggest that MAG potentiates CS via blocking the expression of *eptA*/*arnT*, thereby reducing the cationic modifications on the phosphate moiety of LPS.

### MAG allosterically shuts PmrA/B signaling to modulate LPS modification

**Fig 5:**
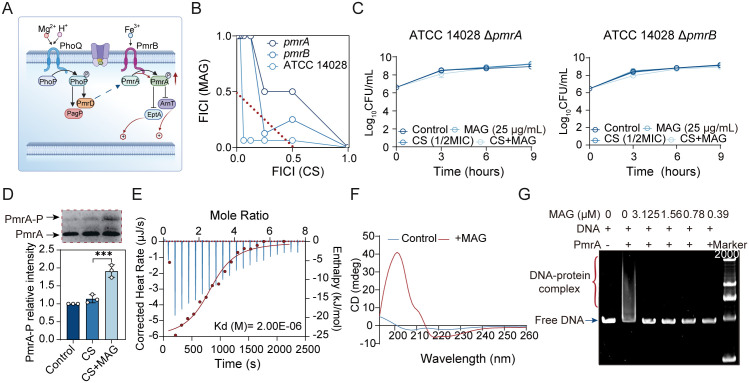
MAG pauses PmrA/B signaling to paralyze bacterial LPS remodeling. **(A)** The scheme for PmrA/B-dependent LPS remodeling (This illustration was created in Biorender. H.R, https://BioRender.com/29k1wd4). **(B)** Genetic deletion of *pmrA* and *pmrB* abolished the synergy between MAG and CS. **(C)** Genetic deletion of *pmrA* and *pmrB* diminishes the synergic bactericidal activity of MAG-CS combination. **(D)** MAG surprisingly promoted the phosphorylation of PmrA regulator protein. **(E)** MAG directly bond to the PmrA regulator protein. **(F)** MAG induced the conformational change of PmrA regulator protein. **(G)** MAG dissociated the cognate DNA from PmrA regulator protein. Assay performed in d was in three biologically independent experiments, and the mean ± s.d. is shown, n = 3. *p < 0.05, **p < 0.01, ***p < 0.001, ****p < 0.0001, determined by nonparametric one-way ANOVA analysis.

As the EptA/ArnT-directed LPS modifications were identified as the determinant enabling MAG to potentiate CS, we next sought to elucidate the target through which MAG regulates the expressions of these genes. In most Gram negative bacteria, the PmrA/B two component system functions as the central hub to modulate chemical modifications of LPS. In this signal transduction system, PmrB acts as the sensor kinase that senses the environmental stimuli and phosphorylates PmrA. Phosphorylated PmrA then directly upregulates the aforementioned genes responsible for LPS modifications (Fig 5A). Therefore, we first speculated that modulation of PmrA/B regulon might drive the MAG-mediated blockade of LPS modifications. To test this hypothesis, the mutants lacking either *pmrA* or *pmrB* were constructed to analyze MAG-CS interaction. As expected, the deficiency of PmrA/B regulon readily abolished the potentiation effect by MAG in both checkerboard (Fig 5B) and time-killing assays (Fig 5C), as sublethal dose of CS (CS: 0.5 µg/mL, 1/2 MIC) was not able to kill the tested mutants as the wildtype. These results implicated that MAG exploits the PmrA/B inactivation to potentiate CS activity in bacteria. Thus, we turn to explaining how MAG halts PmrA/B signaling to affect LPS modifications. As mentioned above, the PmrA/B responds to various exogenous or endogenous stimuli such as cytoplasmic pH, magnesium limitation, and iron availability to regulate the gene expression. However, none of these factors showed significantly changes in bacterial cells treated with or without MAG (S10–S12 Figs). This result indicated that the MAG might target the PmrA/B directly rather than altering their activating stimuli. As shown above, the regulatory control of PmrA/B depends on phosphorylation of PmrA. In this view, the phosphorylation of PmrA was analyzed using Phos-tag gel, where the phosphorylated PmrA would demonstrate a retarded migration at positions of higher molecular weight on the gel compared with its nonphosphorylated counterpart. Surprisingly, the addition of MAG promoted the phosphorylation of PmrA, opposite to the observed phenotype of paused PmrA/B signaling (Figs 5D and S13). This led us to hypothesize that MAG restricts PmrA/B functionality thereby triggering compensatory feedback, partially supported by the increased transcription of both *pmrA* and *pmrB* (S14 Fig). Hence, the isothermal titration calorimetry (ITC) was performed to test the potential interaction between PmrA and MAG. As shown in Fig 5E, MAG displayed high affinity as a ligand to PmrA with the equilibrium dissociation constant (Kd) values of 2.0 μM. Supporting this observation, circular dichroism (CD) spectroscopy analysis revealed the binding of MAG induced conformational change of PmrA regulator protein (Fig 5F). The results indicated the major changes occurs in the altered β-sheets (S15 Fig). Given that β-sheets are the important structural constitutes of DNA-binding domain (DBD) of PmrA [29], it is possible that the binding of MAG obstructs the interactions of PmrA with DNA. Hence, we employed a modified electrophoretic mobility shift assay (EMSA) to clarify whether MAG affects the functions of PmrA by dissociating the PmrA from its target promoters. Using a binding motif from *eptA* promoter as an indicator, it was found that the addition of MAG overwhelmingly reduced the DNA binding ability of PmrA, leaving most portions of DNAs in free form (Fig 5G). Together, these data corroborated that MAG allosterically dissociated the PmrA from its target DNA to pause gene expressions that responsible for LPS modification, thereby potentiating CS binding and killing actions.

### MAG-CS combination as a viable therapeutic regimen against infection *in vivo*

**Fig 6:**
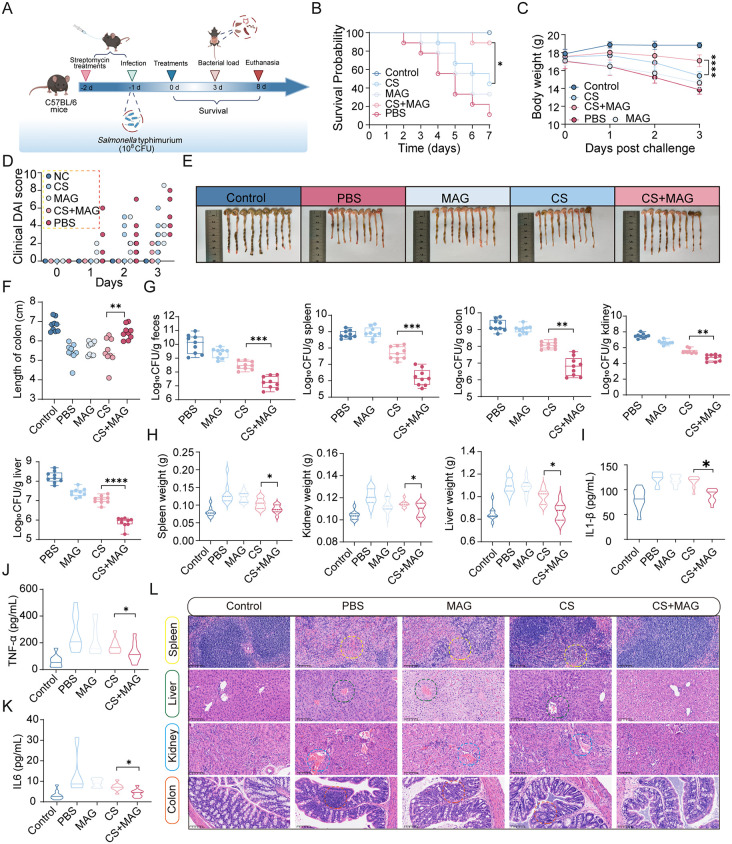
MAG-CS combination as a viable therapeutic regimen against infection *in vivo.* **(A)** Scheme of experimental procedure of animal trial, where the animals (n = 9) were infected via oral gavage of lethal dose of *Salmonella* at 10^8 CFU/ mouse, then treated by CS (5 mg/kg), MAG (50 mg/kg) or CS-MAG (5 + 50 mg/kg). The bacterial loads in infected mice were examined at 3 days post infection and the survival of mice was monitored until 8 days post infection. The separate groups were used for survival and bacterial load experiments (This illustration was created in Biorender. H.R, https://BioRender.com/6yzdbao). **(B)** Survival curve of animals received CS, MAG and their combinations. **(C)** Body weights of infected mice with treatments of PBS, CS, MAG and MAG-CS combination. **(D)** Clinical DAI scores (integrates weight loss, diarrhea, reduced activity and hunched posture) were assessed daily for 3 days; Colon length at indicated day post infection (**E**) and the quantitative analysis **(F)**. **(G)** Bacterial load in the feces, spleen, colon, kidney, liver of infected mice treated by CS, MAG, and their combinations. **(H)** The weights of major infected organs (spleen, kidney and liver) at indicated day post infection. **(I-K)** The production of pro-inflammatory TNF-α, IL-1β and IL-6 in infected mice treated by CS, MAG, and their combinations. **(L)** Histological tests of infected mice treated by CS, MAG, and their combinations. All animal trials performed in C-L were shown as mean ± s.d of data from all animals. *p < 0.05, **p < 0.01, ***p < 0.001, ****p < 0.0001; determined by nonparametric one-way ANOVA analysis.

On account of promising synergistic action between MAG and CS, it is of importance to evaluate the efficacy of their combination *in vivo*. To this end, a classic murine infection model was established as illustrated in Fig 6A. The mice were infected by lethal dose of *S*. Typhimurium via oral gavage after streptomycin pre-treatment. After receiving CS, MAG or the CS-MAG combination via i.p., both survival and bacterial burdens were assessed as clinic outcomes. Throughout the experiment, mice receiving the MAG-CS combination exhibited a markedly improved survival rate (45% or 56%), outperforming either CS or MAG alone (Fig 6B). In addition, compared to mice received CS or MAG alone, their combination also markedly mitigated the body weight losses (Fig 6C) and disease activity (Fig 6D) caused by infection. As a major hallmark of *Salmonella* infection, the pronounced colon shortening was observed in infected mice, which partially restored by CS monotherapy but more effectively alleviated by MAG-CS combination (Fig 6E and 6F). As to pathogen clearance, the combination therapy based on MAG and CS markedly reduced the bacterial loads in feces, liver, kidney, colon and spleen by up to two orders of magnitude (Fig 6G). In concert with these observation, the weights of infected organs were also found to be reduced by MAG-CS combination (Fig 6H), likely reflecting resolution of inflammation by the combination therapy. This interpretation was further supported by significantly lowered inflammatory cytokines detected in serum of MAG-CS treated mice (Fig 6I–6K). As revealed by histological analysis, the combination therapy provided maximal protection against *Salmonella*-induced tissue damage, preserving structural integrity and reducing necrosis compared with monotherapy (Fig 6L).

**Fig 7:**
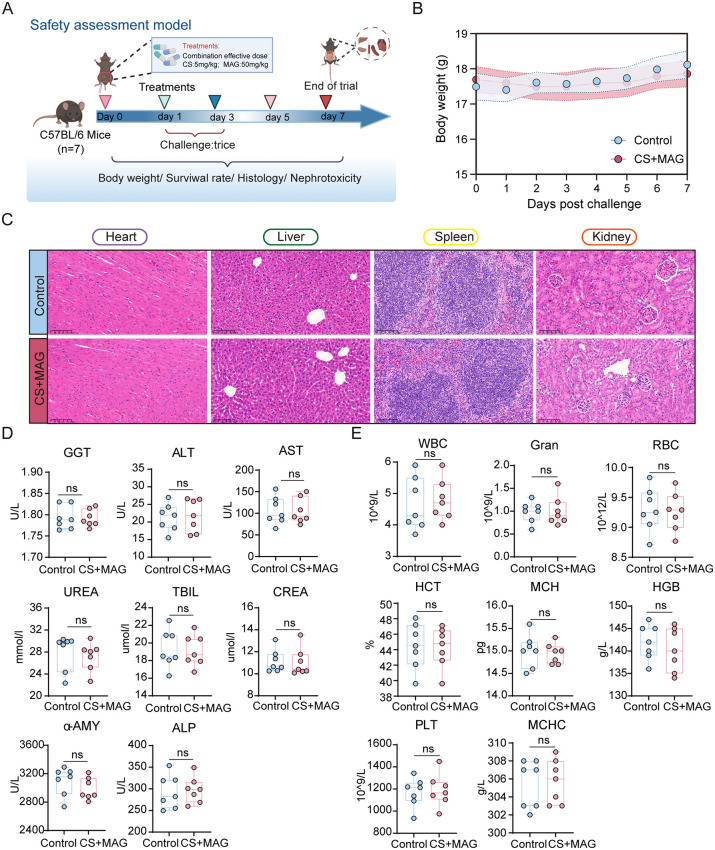
Biosafety analysis of MAG-CS combination *in vivo.* **(A)** Scheme of experimental procedure of animal trial for biosafety analysis (n = 7, This illustration was created in Biorender. H.R, https://BioRender.com/brf7o52). **(B)** Body weight of mice (n = 7 per group) received PBS (control, 100 µL) and CS-MAG (5 + 50 mg/kg, equals to the therapeutic dose). **(C)** H&E staining of hearts, livers, spleens and kidneys from mice (n = 7 per group) received PBS (control, 100 µL) and CS-MAG (5 + 50 mg/kg, equals to the therapeutic dose). **(D)** Serum indices of mice (n = 7 per group) received PBS (control, 100 µL) and CS-MAG (5 + 50 mg/kg, equals to the therapeutic dose), GGT: Gamma-glutamyltransferase, ALT: Alanine Transaminase, AST: Aspartate Transferase, TBILL: Total Bilirubin, UREA: Urea, ALP: Alkaline Phosphatase, CREA: Creatinine, α-AMY: α-Amylase. **(E)** Complete blood counts of mice (n = 7 per group) received PBS (control, 100 µL) and CS-MAG (5 + 50 mg/kg, equals to the therapeutic dose), WBC: White blood cell, Gran: Granulocyte, RBC: Red blood cell, HGB: Hemoglobin, HCT: Hematocrit, MCH: mean corpusular hemoglobin, MCHC: mean corpuscular hemoglobin concentration, PLT: platelet count. All animal trials performed in D&E were shown as mean ± s.d of data from all animals. *p < 0.05, **p < 0.01, ***p < 0.001, ****p < 0.0001, determined by non*p*arametric one-way ANOVA.

In addition to its *in vivo* efficacy against infection, the biosafety of CS-MAG combination was evaluated (Fig 7A). No mortality or significant changes in body weight were observed in mice treated by CS-MAG combination throughout the trial (Figs 7B and S16). Histological analysis of major organs, including the heart, liver, spleen, and kidney, revealed normal tissue morphology following CS-MAG combination administration (Fig 7C). To address the concern on nephrotoxicity of colistin-dependent treatment, complete blood counts and serum biochemical parameters were assessed and found to remain within physiological ranges, indicating that CS-MAG combination elicited no detectable systemic or organ toxicity (Fig 7D and 7E). Collectively, these *in vivo* data demonstrate that MAG enhances therapeutic efficacy of CS without detectable adverse effects, underscoring its potential as a safe and effective strategy to combat pathogenic infections in clinic.

## Discussion

**Fig 8:**
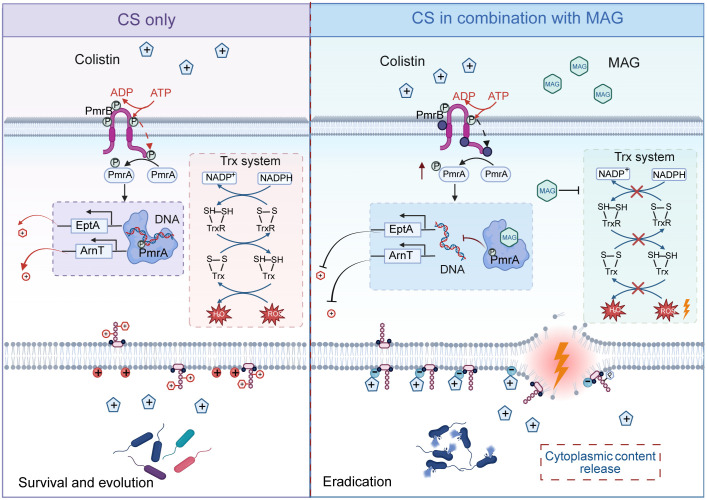
Mode of action of MAG to potentiate CS activity. (This illustration was created in Biorender. H.R, https://BioRender.com/qa4fj87).

The insufficiency in antimicrobial development has been neglected to some extent amid the upstaging of other pandemics such as COVID-19 [30]. However, infections caused by bacteria especially drug-resistant pathogens still remain among the deadliest in clinical settings [31]. The fundamental problem lies in the lack of interest for drug industries in developing novel antimicrobial therapeutics due to extremely high expenditure and limited economic incentives [32,33]. To better balance the void in the antimicrobial pipeline with the risky investment required for new drugs, using antibiotic adjuvants to reinvigorate the existing antimicrobial agents appears to be one of the most feasible approaches [34]. To this end, this study established a high-throughput system to uncover the potential compounds capable of rejuvenating CS, a last-resort antibiotic. Among the screened chemicals, a lignan compound, MAG, was identified as an interesting lead that enhances CS efficacy. Lignans belong to diphenolic class that naturally-occurring in many plants and have been frequently found to actively modulate host responses to oxidative stress, neurodegeneration, tumorigenesis and infection [35]. Lignans exemplified by MAG have been severally reported to suppress the Gram-positive pathogens by inhibiting the *Trx* system [36]. However, the Gram-negative bacteria exploit the glutathione (GSH) systems to offer additional antioxidant capacity, which compensates impaired redox balance in *Trx* deficiency and undermines the potency of compounds like MAG [37]. Thus, the potential of MAG to combat antibiotic resistances in Gram negative bacteria has been long overlooked. In this study, we propose the PmrA/B signaling as a new target of MAG that has not been previously mapped. The PmrA/B signaling is generally responds to environmental changes in ions such as iron, magnesium and proton and initiates the transcriptional programs that modify the bacterial envelope for counteraction [38]. In our previous work, 7,8-dihydroxyflavone, myricetin, and luteolin were found to restore CS activity by targeting the PmrA/B system [39]. These three natural flavonoids disrupt the bacterial iron homeostasis to interfere the iron-sensing module of PmrA/B and thereby constitutively disarming its activation for bacterial membrane modification. Although sharing the same target, the MAG elicits a new mechanism to pause PmrA/B signaling by directly acting on the response regulator PmrA. Through binding to PmrA protein, the MAG dissociates the promoters of LPS-modifying genes from the PmrA, even when PmrA/B is in its activated form (Fig 8). In light of target vulnerability which defined as the level and magnitude of drug-target engagement required to generate a pharmacological response [40], it is conceivable that perturbing the regulatory functionality of PmrA/B may represent a more vulnerable strategy than inhibiting its activation. This is because, as indicated by previous study, the activation and magnitude of PmrA/B-dependent LPS modification varies across bacterial species and biological contexts [41]. Therefore, interference with PmrA/B transcriptional output is more favorable as this directly leads to stalled downstream effects on gene expression and phenotype.

Some microbial species harness an inherent capability to adaptively resist certain antibiotics through transient responses under given conditions [42]. This is outlined as adaptively intrinsic antibiotic resistance which often counteractively reduce antibiotic efficacy but has not received sufficient attentions. As a result, increasing numbers of failures in antibiotic therapies predicted by standard AST have been reported due to intrinsic resistance of bacteria in the host. For instance, Band and colleagues reported that AST-recommended antibiotics were unable to eliminate *Enterobacter cloacae* infection in sepsis model because intrinsic resistance was triggered by the host innate immune defense [43]. These data indicate that the widely applied standard medium may be challenged to accurately reflect the responses of antimicrobial therapy *in vivo* the recapitulating the infection sites [44]. To cope with this, the present study employed a medium mimicking the microenvironment of phagocytic cells, in which pathogens like *S.* Typhimurium already display high-level resistance to cationic antibiotics. In this screening, MAG was found to potentiate CS efficacy under host-like conditions rather than in rich medium, suggesting its unique ability to selectively repress intrinsic resistance. This trait endows MAG with the expected activity to enhance the CS potency at *in situ* niches there pathogen replicate and through which they translocate. Consequently, the MAG-CS combination not only reduced bacterial propagation in intestinal tract but also predominantly obstructed the bacterial translocation to extraintestinal sites such as the liver, kidney and spleen. A very recent report revealed that the treatments with bactericidal antibiotic generally induces more proinflammatory cytokines because the pathogens in bloodstream release highly immunogenic eDNA to engage the host immune response under bactericidal agents [45]. Nevertheless, the current study showed that the incorporation of MAG also reduced the proinflammatory cytokine secretion in serum during antibiotic treatment. We reasoned that this is because MAG potentiates the CS to selectively reduce the total bacterial biomass within the immune cells, by which subsequently moderating the release of immunogenic bacterial components. Given that MAG has been reported to modulate inflammation in various pathogeneses [46,47], it is also possible that the MAG exerted anti-inflammatory activity per se. Considering the multifaceted role of MAG in modulating antibiotic treated infection outcomes, it is of great interest to further explore the understudied versatility of such agents for combating infections caused by resistant pathogens.

As one of the last-resort antibiotics, CS was only sparingly used in the last century due to its nephrotoxicity, yet it resurged into clinics to control multidrug-resistant Gram negative bacteria especially carbapenemase-producing Enterobacteriaceae [48,49]. Soon after the revival of CS as a frontline therapy, the phenotypic and genotypic resistances to CS were frequently detected in isolates from diverse origins [50]. The situation was further exacerbated by the emergences of plasmid-mediated *mcr* (mobilized colistin resistance) genes, first identified by Liu et al. in *E. coli* and subsequently found in many other species [51,52]. This transferable mechanism has been regarded as a major driving force and genetic determinant of CS resistance and has been at the epicenter of AMR research for years [53]. It is noteworthy on the premise that *mcr*-encoded phosphoethanolamine transferases mediate only moderate resistance level and often incur a fitness cost to bacteria [54–56]. This was observed the present study, where an isolate bearing a *mgrB* mutant exhibited higher CS resistance than an isolate with carriage of *mcr*-positive plasmid. Accordingly, this study included CS-resistant isolates with both plasmid- and chromosome-mediated CS resistance for test. The current results indicated that MAG was able to restore the CS potency on such strains, but the MAG was less active against the *mgrB*-mutant strain. This can be explained by the loss of stringent control of PhoP/Q in the *mgrB* mutant, which induces over-activation of the PmrA/B system, partially offsetting MAG’s inhibitory effect. Previous reports have extensively documented that bacteria are prone to build up resistance under selection of bactericidal agents like CS [57]. However, this resistance evolution was completely suppressed by the addition of MAG. We propose two possible explanations for this observation. First, in alignment with a prior study, MAG accelerated CS killing to promptly reduce viable cells and thereby limit opportunities for mutation accumulation [58]. Second, MAG augmented the CS-induced toroidal pore formation, leading to rapid loss of key cellular components that necessary for evolution, such as DNA and proteins (including the polymerases, helicase and recombinase that might be important for error-prone repair). This depletion may underlie the observed paralysis of evolutionary adaptation under CS stress.

## Conclusion

In summary, this study identified a lignan compound, MAG, as an active adjuvant that potentiates CS activity under host-mimicking conditions. MAG was elucidated to pause PmrA/B-mediated transcriptional control by dissociating PmrA regulator from its target DNA. This action reduces PmrA/B-dependent LPS remodeling to enhance CS binding and killing. The present work highlights the MAG-CS combination as a viable therapeutic regimen with genetically-defined mechanism to accelerate pathogen clearance and proposed the transcription factor decoy as a feasible approach to inactivate two component systems such as PmrA/B.

## Methods

### Ethic statement

The present study was carried out from 2024 (the infection trial, Jun to Oct) to 2025 (*In vivo* toxicity evaluation, Nov) at laboratory animal center of South China Agricultural University in accordance with its recommendations of the ethical guidelines. All animal experimental protocols were reviewed and approved by the South China Agricultural University Institutional Animal Ethics Committee (Approval no.: 2025c133).

### Bacterial strains and media

In this study, the model strains and their gene-knockout mutants used for experiments were listed below in Table 1. The colistin-resistant isolates were from the samples collected during 2021–2023. Bacteria were propagated in Luria-Bertani (LB) media. For all tested strains, antimicrobial susceptibility was determined by host-mimicking medium (LPM) as described previously [39].

### Genetic manipulation of strains

All primers used in this study were listed in S1 Table. The *pmrA*, *pmrB*, *eptA*, and *arnT* genes knockout mutant strains were made by λRed recombination system described previously [59]. The constructs of P_*arnT*_-Lux, P_*eptA*_-Lux and pBAD24::*pmrA*-HA were generated as described in previous study [39]. To express the PmrA protein in prokaryotic cells, *pmrA* with a 6 × His tag was cloned into the vector PET-28a to generate the pET-28a-*pmrA*, which was then transformed into *E. coli* BL21(DE3) for recombinant protein expression.

### Screening procedure of CS potentiators

A total of 500 chemicals from the lab collection were subjected to screening using an in-house protocol based on previously published method [22,60]. In brief, a total of 8 µg/mL of CS (1/4 MIC in LPM) was combined with 50 µM of each chemical from the collection and their interaction was interpreted by the ε̃ value. The ε̃ was calculated by the equation: ε̃ = (*W*_XY_ − *W*_X_*W*Y)/|W̃ _XY_ − *W*_X_*W*Y|, where W̃ _XY_ = min [*W*_X_, *W*_Y_] if *W*_XY_ > *W*_X_*W*_Y_ and is 0 otherwise. The *W*_X_, *W*_Y_ and *W*_XY_ stand for normalized reduced growth rate caused by the drugs (*W*_X_ = *g*_X_/*g*_*φ*_, *W*_Y_ = *g*_Y_/*g*_*φ*_, *W*_XY_ = *g*_XY_/*g*_*φ*_), where *g*_X_, *g*_X_, *g*_XY_ and *g*φ are the measured *g*rowth rates with single drug, drug combination and with no drug. If W̃_XY_ was numerically greater than min [W_X_, WY], then ε̃ was equal to {(W_XY_ − min [W_X_, W_Y_])/(1 − min [W_X_, WY]) + 1. When ε̃ falls between -1 and -0.5, the result was considered synergy; otherwise, interactions were categorized as either additive or antagonistic.

### Antimicrobial susceptibility and drug interaction analysis

Susceptibility test was performed according to Clinical and Laboratory Standards Institute (CLSI) standards. Briefly, the indicated drugs were serially diluted into the bacterial suspension of ~10^6 colony-forming units (CFU)/mL and cultivated for 18 hours 37°C. The minimum inhibitory concentration (MIC) of antibiotics were defined as the lowest concentrations of an antimicrobial agent that results in the complete inhibition of bacterial growth [61]. The interaction of drug combination was first validated using Synergyfinder 3.0, where the calibrated bacterial growth upon CS-MAG combination at different doses were used to generate high-resolution synergy landscape [23]. Then, the strain-specific drug interaction was then quantified by fractional inhibitory concentration index (FICI) calculated as follows: FIC index = MIC_ab_∕MIC_a_ + MIC_ba_∕MIC_b_ = FIC_a_ + FIC_b_. The FICI ≤ 0.5 indicates synergy, FICI > 0.5 but ≤ 2.0 suggests an additive effect, and FICI > 2.0 indicates antagonism, the results were interpreted by either isobologram or chequerboard analysis.

### Time-dependent bactericidal assay

Bacterial suspensions were diluted to a concentration of approximately 10^6^ CFU/mL in LPM medium. Subsequently, each aliquot was exposed to designated concentrations of CS (1/2 MIC), MAG (25 μg/mL), or their combination. Survivors were determined at various time intervals of 0, 3, 6, and 9 hours by plating on the selective agar [39].

### Resistance evolution experiment

The resistance evolution experiment was performed following previously described protocol [62]. Briefly, the strain 14028s was cultured in fresh LPM medium until logarithmic stage. Subsequently, the cultures were transferred to fresh LPM medium containing sub-lethal CS (1/4 MIC) with or without MAG. The mixture was then incubated at 37°C with shaking at 180 rpm for 24 hours. Over the course of 21 days, the cultures were continuously subcultured, with the MIC values of evolved bacterial subpopulations being monitored throughout this period. The evolved bacteria were subjected to whole-genome sequencing to elucidate the mutations and the genome data were deposited in the public databased under accession number PRJNA1372104.

### Assessment of intracellular ROS

The assay for intracellular ROS production was conducted in accordance with a method previously reported [63]. Bacteria were exposed to designated concentrations of CS (16 μg/mL), MAG (25 μg/mL), or their combinations. Following a 30-minute incubation with an ROS-sensitive dye (DCFH-DA, 10 μM, Beyotime, China), the fluorescence intensity was quantified using a microplate reader (PerkinElmer, USA) at an excitation wavelength of 488 nm and an emission wavelength of 525 nm.

### Cell membrane permeability test

Membrane permeability was carried out according to a protocol outlined in a previous study [64]. Briefly, the bacteria were initially exposed to CS (16 μg/mL) with or without MAG (25 μg/mL) at 37°C for 30 minutes. Subsequently, samples were treated with the fluorescent dye propidium iodide (PI, 30 μM, Beyotime, China) or 1-N-phenylnaphthylamine (NPN, 10 μM, Meilunbio, China) in the dark for 30 minutes. The fluorescent signals were quantified using a microplate reader at excitation/emission wavelengths of 535/617 nm (PI) or 350/420 nm (NPN). Each test was conducted in triplicate.

### Cytoplasmic profiling of bacteria under treatments

The bacterial cytoplasmic profiling was performed in accordance with prior protocol [65]. Briefly, overnight cultures of bacterial were diluted at 1:100 into fresh LPM medium and cultivated as specified to achieve a bacterial suspension with a density of 10^8 CFU/mL. Bacteria were then exposed to CS (8 μg/mL) with and without MAG (25 μg/mL) for a duration of 2 hours, subjected to three washes with cold PBS, and then resuspended. Subsequently, a combination of FM4–64 (2 μg/mL, Thermo Fisher, USA) and DAPI (3 μg/mL, Thermo Fisher, USA) was added into the suspension, followed by a 30-minute incubation in darkness. Lastly, the bacteria were washed with PBS and resuspension were visualized using a super-resolution confocal microscope, the TCS SP8 STED 3X (LEICA, Germany).

### Cytoplasmic leakage assay

Cytoplasmic leakage was measured following a previously published protocol [66]. Briefly, bacteria in the exponential phase were resuspended in LPM medium and exposed to CS (16 μg/mL) with or without MAG (25 μg/mL) for a period of 4 hours. Following this treatment, the supernatant was then collected by centrifugation at 6000 rpm for 5 minutes, and the concentrations of extracellular DNA and proteins were quantified using a spectrophotometer. In a separate experiment, bacteria in the logarithmic growth phase were similarly resuspended in LPM medium lacking K^+^ and subjected to the aforementioned treatment. Extracellular concentration of potassium ions was determined using a flame photometer FP6410 (LABO-HUB, China).

### BODIPY-TR cadaverine displacement assay

Overnight cultures of bacteria were diluted at a 1:100 into fresh LPM medium and incubated for 24 hours. Subsequently, MAG (25 μg/mL) was added, and cultures were incubated at 37°C with shaking at 180 rpm for a duration of 6 hours. Following centrifugation to remove the supernatant, bacterial pellet underwent a single wash with Tris-HCl, followed by resuspension. The BODIPY-TR Cadaverine probe (MCE, USA) was added, and suspensions were subjected to incubation at room temperature in darkness for 4 hours. Bacterial cells were then harvested via centrifugation, washed with LPM, resuspended, and transferred to a black 96-well plate, with 100 µL loaded in each well. CS (8 μg/mL) were introduced into each well and incubated at 37°C for 1 hour. Fluorescence was measured at 580/620 nm using a multifunction microplate reader (PerkinElmer, USA) [67].

### Membrane charge analysis

Bacteria were cultivated overnight and, then transferred into fresh LPM medium. Then t cultures were either treated by CS (8 μg/mL), MAG (12.5 μg/mL) or the combination of both at 37°C for 4–6 hours. Aliquot was washed by distilled water for resuspension. Finally, the membrane charges of the bacteria were assessed utilizing a zeta potential analyzer [68].

### Membrane lipid profiling

Bacterial membrane lipid modifications were analyzed by extracting lipid A of bacteria using a previously-described method with slight modifications [69]. Briefly, the bacteria was cultured at 37°C overnight in LPM medium with or without MAG (50 μg/mL), then were subsequently resuspended in chloroform: methanol:aqueous solution (1:2:0.8), and breaked. Following centrifugation at 2000 rpm for 30 minutes, the cell pellet was resuspended in natrium aceticum. The suspension was then added to a biphasic solution with chloroform/methanol/water ratio of 2:2:1.8 and transferred to a spin vial for evaporation. Extracted lipids were resuspended in chloroform/methanol (4:1, v/v) and dried with nitrogen gas before lipid analysis using MALDI-TOF.

### Gene transcription analysis

Gene transcriptions of bacteria under treatments were mainly analyzed by RT-qPCR and transcriptional reporter assays. The RT-qPCR was performed as previously documented. In brief, the total RNA was extracted using the Total RNA Kit I (Omega, China) and reverse-transcribed into cDNA. The qRT-PCR was conducted with 16S ribosomal RNA as the normalization control. The primers employed for RT-qPCR were delineated in S1 Table. Each experiment was conducted in triplicate. Transcriptional reporter assay was performed as described previously [39]. In brief, a pUC-luxCDABE plasmid was transformed into the bacterial strain, then the as-prepared strain, which was then cultivated in presence of CS (1 μg/mL) and MAG (6.25 μg/mL) in LPM for 6 hours. The gene transcription were analyzed by the spectrophotometry.

### Phos-tag assay

Phosphorylation of PmrA regulator protein was detected by the Phos tag assay as previously described [39]. In brief, 14028s/pBAD-*pmrA*-HA was cultivated in LPM containing 5 mg/mL arabinose until reaching the logarithmic phase. Then, the cells were exposed to CS (2 μg/mL) with or without MAG (6.25 μg/mL), for 1.5 hours, followed by PBS washing for 3 times. Then the cell pellets were lysed and the resulting lysates were subsequently denatured by admixing with SDS buffer. After denaturation, each aliquot was loaded onto a Phos-tag gel where the phosphyrylated and unphosphorylated proteins were separated by electrophoresis and subsequently transferred to polyvinylidene difluoride for immunoblotting. The blots were probed with indicated antibodies, and the phosphorylation level was determined with ImageJ.

### Expression and purification of PmrA protein

The BL21/pET-28a:: *pmrA*-his6 plasmid was constructed for expression and purification of PmrA. The constructed strain was added to induce PmrA expression by incubating in fresh LB medium with shaking at 200 rpm. Once the OD_600_ reached 0.5, 0.2 mM IPTG was employed to induce the protein expression at 16°C for an additional 16–20 hours. Thereafter, PmrA was purified by nickel affinity chromatography, in accordance with prior publications [70].

### Isothermal titration calorimetry (ITC)

Binding affinity of PmrA protein with MAG was evaluated by ITC (TA Instruments, USA), with the drug solubilized in DMSO. Data were analyzed with the instrument’s dedicated software to determine equilibrium dissociation constants, following established protocols [71].

### Circular dichroism (CD) spectra analysis

CD spectra of PmrA with or without MAG (6.25 μg/mL) were analyzed by a CD spectrophotometer (Chriascan, UK) with the scanning wavelengths ranged from 190 to 260 nm. The CDNN software was used to calculate secondary structure fractions based on established protocol [72].

### Electrophoretic mobility-shift assay (EMSA)

EMSAs were performed as described with minor modified as follows [73]. In brief, the PmrA protein was pre-incubated with MAG at room temperature for 1 hour, followed by addition of synthesized oligonucleotides containing the PmrA-binding motif in a 10 μL reaction system (2 μL of Binding Buffer, 2 μL of ddH_2_O, 4 μL of protein, and 2 μL of DNA). The mixture was incubated in obscurity at 25°C for 20 minutes, followed by the addition of 2 μL of 6 × loading buffer. Samples were resolved by non-denaturing electrophoresis in 0.5 × TBE buffer (120 V, 1 hour), stained with SYBR Green at 4°C for 20 minutes, and visualized.

### Animal experiments

A standard Salmonella infection model was constructed as previously described [74]. Female C57BL/6 mice at 8 weeks of age were randomly divided into five groups and orally infected with 10^8^ CFU of strain 14028s. Then the infected mice received treatments including CS (5 mg/kg), MAG (50 mg/kg), CS + MAG (5 + 50 mg/kg) and PBS (n = 9 for each group) at one day post infection through intraperitoneal (i.p.) route (all applied therapeutics were given to animals at 100 μL where the CS was dissolved in sterile saline and the MAG was dissolved in DMSO then suspended in corn oil). At 3 days post infection, the bacterial burdens in the organs (the liver, colon, spleen, and kidney) and fecal samples were enumerated. Meanwhile, the pathogenesis of infected mice after each treatment were evaluated by the clinical disease activity (details provided below), colon length and histopathological analysis. The survival and body weights of mice in each group were monitored daily throughout the experiments.

### Clinical disease activity

To comprehensively evaluate the efficacy of each treatment, the clinical disease activity index (DAI) was introduced following protocol previously described [75]. In brief, the DAI was assessed based on weight loss, diarrhea (stool consistency), animal activity and posture: 1) Body weight loss was scored as 0 for ≤5%, 1 for 5–14%, 2 for 15–19%, and 3 for ≥20% losses; 2) Stool consistency was scored as follows: 0, well-formed pellets; 1, semi-formed stools; 2, soft stools; and 3, liquid stools adhering to the anus. 3) Activity was assessed as 0, normal; 1, fully mobile but less active than usual; 2, occasional movement with response to prompting; and 3, minimal movement with poor responsiveness. 4) Posture was scored as 0, normal; 1, slight hunching; 2, pronounced hunching with intermittent recovery; and 3, sustained hunching without recovery.

### *In vivo* toxicity evaluation

*In vivo* toxicity of combination was performed according to previous method [13]. 7-week-old female C57BL/6J mice were randomly allocated to seven ventilated cages (7 mice/ cage). A 3 day acclimatization period was observed prior to conducting the test. On challenge day, mice received either 100 µL of PBS (control) or a mix of CS (5 mg/kg) and MAG (50 mg/kg), both in 100 µL. Their body weights and survival rates were tracked daily for seven days. On the 8^th^ day post challenge, the mice were euthanized to collect heart, spleen, kidney and liver, which were used for for pathological analysis using H&E staining. The serum indices and complete blood counts tests were performed on the blood collected from the mice.

### Statistical analysis

Results are expressed as means ± SD, and all experiments were carried out in triplicate unless indicated otherwise. The results were statistical analysis was performed using GraphPad Prism 9 software. Unless stated otherwise, the statistical significance of comparison was assessed using the unpaired t-tests or one-way analysis of variance (ANOVA) (*P < 0.05, **P < 0.01, ***P < 0.001, and ****P < 0.0001). All figures were assembled using Adobe Illustrator.

## Supporting information

S1 Fig Chemical structure of MAG. (TIF)

S2 Fig Synergistic interaction between MAG and CS on CS-sensitive Gram negative bacteria in LPM medium. *S.* Typhimurium ATCC14028 (A), *E. coli* ATCC25922 (**B**), *K. pneumoniae* ATCC700603 (**C**).
(TIF)

S3 Fig Isobolograms of the combination of CS and MAG against different CS-sensitive model strains. (TIF)

S4 Fig Synergistic interaction between MAG and CS on S.Tm str. 14028s in standard rich medium (MH medium). Checkerboard illustration (**A**); Isobologram analysis (**B**).
(TIF)

S5 Fig The dose-response fitting of synergistic interaction between CS and MAG on CS-resistant isolates. *S.* Typhimurium (17ES) (A), *E. coli* (2012FS) (B), *K. pneumoniae* (CMG) (C).
(TIF)

S6 Fig The synergistic interaction between MAG and CS on CS-resistant isolates indicated by the checkerboard assays. *S.* Typhimurium (17ES) (**A**), *E. coli* (2012FS) (**B**), *K. pneumoniae* (CMG) (**C**).
(TIF)

S7 Fig Mutations found in the genome of bacteria exposed to CS alone during serial passage. (TIF)

S8 Fig Checkerboard assay to elucidate CS potentiation by MAG was not fully dependent on MAG-mediated *Trx* insults. *trxB* deficiency reduced yet not abolished synergistic interaction between MAG and CS **(A)**; Exogenous addition of ROS scavenger MnTBAP reduced but not diminished the synergy between CS and MAG (B).
(TIF)

S9 Fig Transcriptional reporter assay showed that the expression of *eptA* and *arnT* were suppressed by exposure to MAG. (TIF)

S10 Fig The pH of bacteria remained unaffected following MAG treatment. (TIF)

S11 Fig MAG treatment did not change the cytoplasmic Mg^2+^ concentration in *S*.Tm str. 14028s. (TIF)

S12 Fig MAG-mediated potentiation was not dependent on modulation on iron signaling. Isobolograms of the CS and MAG combination against *tonB*-/*feoB*-deficient mutants (**A**), The introduction of exogenous ferric iron did not abolish the synergistic effect between CS and MAG (**B**).
(TIF)

S13 Fig Uncropped membrane image for Phos-tag assay relative to Fig 5D. (TIF)

S14 Fig Expression of pmrA and pmrB were primed in S.Tm str. 14028s upon exposure to MAG. (TIF)

S15 Fig The CD analysis revealed that MAG induced conformational changes of PmrA regulator protein. (TIF)

S16 Fig Survival curves of animals received CS-MAG combination or PBS control. (TIF)

S1 Table Oligonucleotides primers used in the current study. (DOCX)

S1 Data Source Data. (XLSX)
