# Supplementary figures and images for: A lignan compound regulates LPS modifications via PmrA/B signaling cascades to potentiate colistin efficacy in vivo
Source: PLoS Pathog. 2025 Dec 29;21(12):e1013843. doi: 10.1371/journal.ppat.1013843 (PMC12774351; doi:10.1371/journal.ppat.1013843)

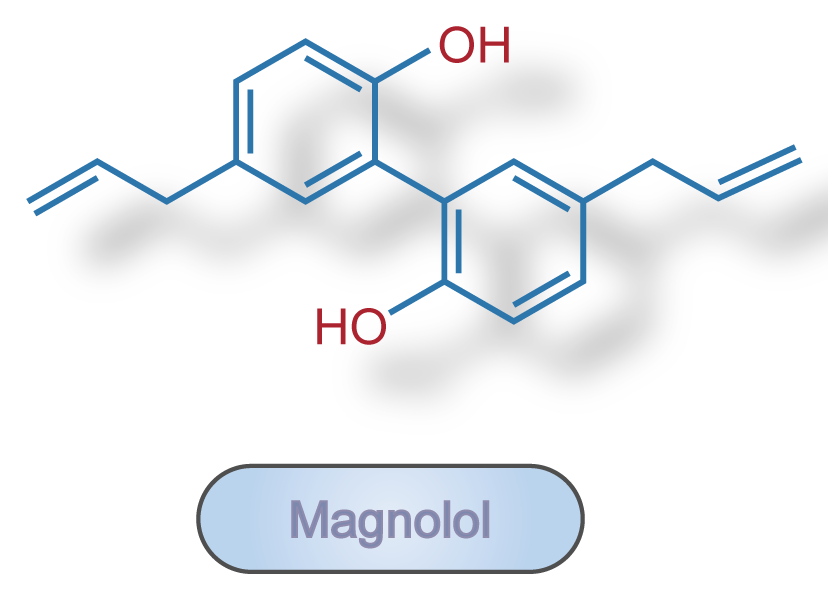

Supplement: S1 Fig — (TIF) [file ppat.1013843.s001.tif]

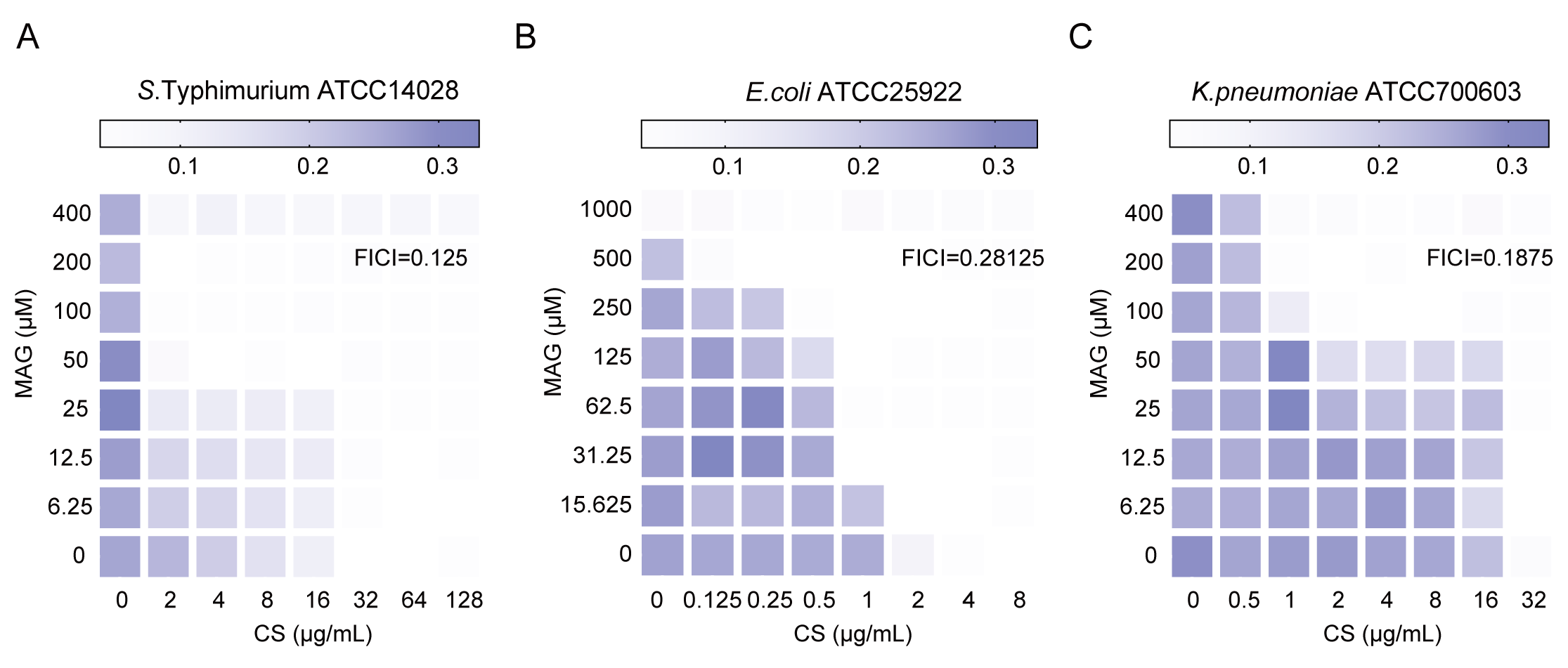

Supplement: S2 Fig — S. Typhimurium ATCC14028 (A), E. coli ATCC25922 (B), K. pneumoniae ATCC700603 (C). (TIF) [file ppat.1013843.s002.tif]

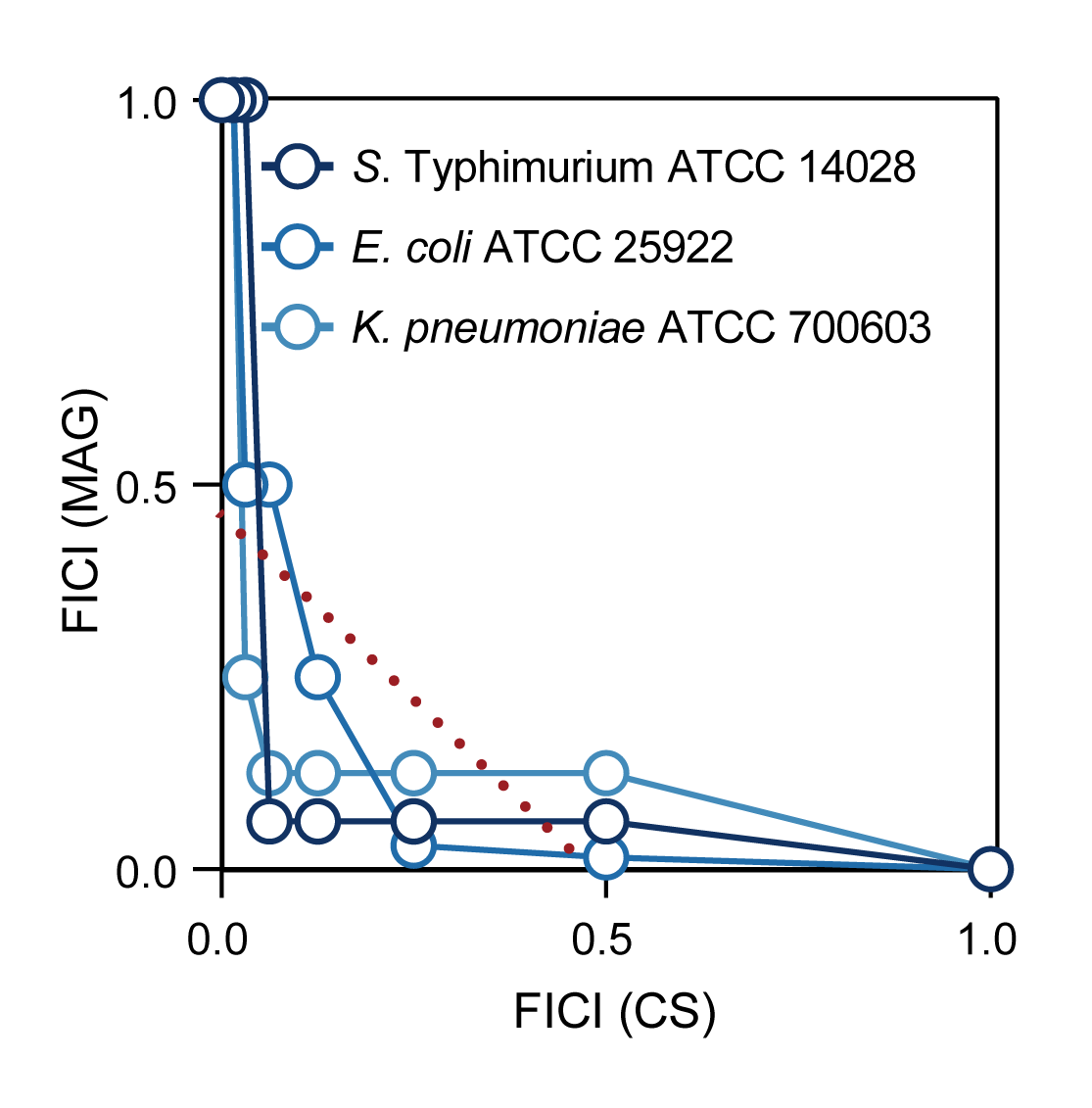

Supplement: S3 Fig — (TIF) [file ppat.1013843.s003.tif]

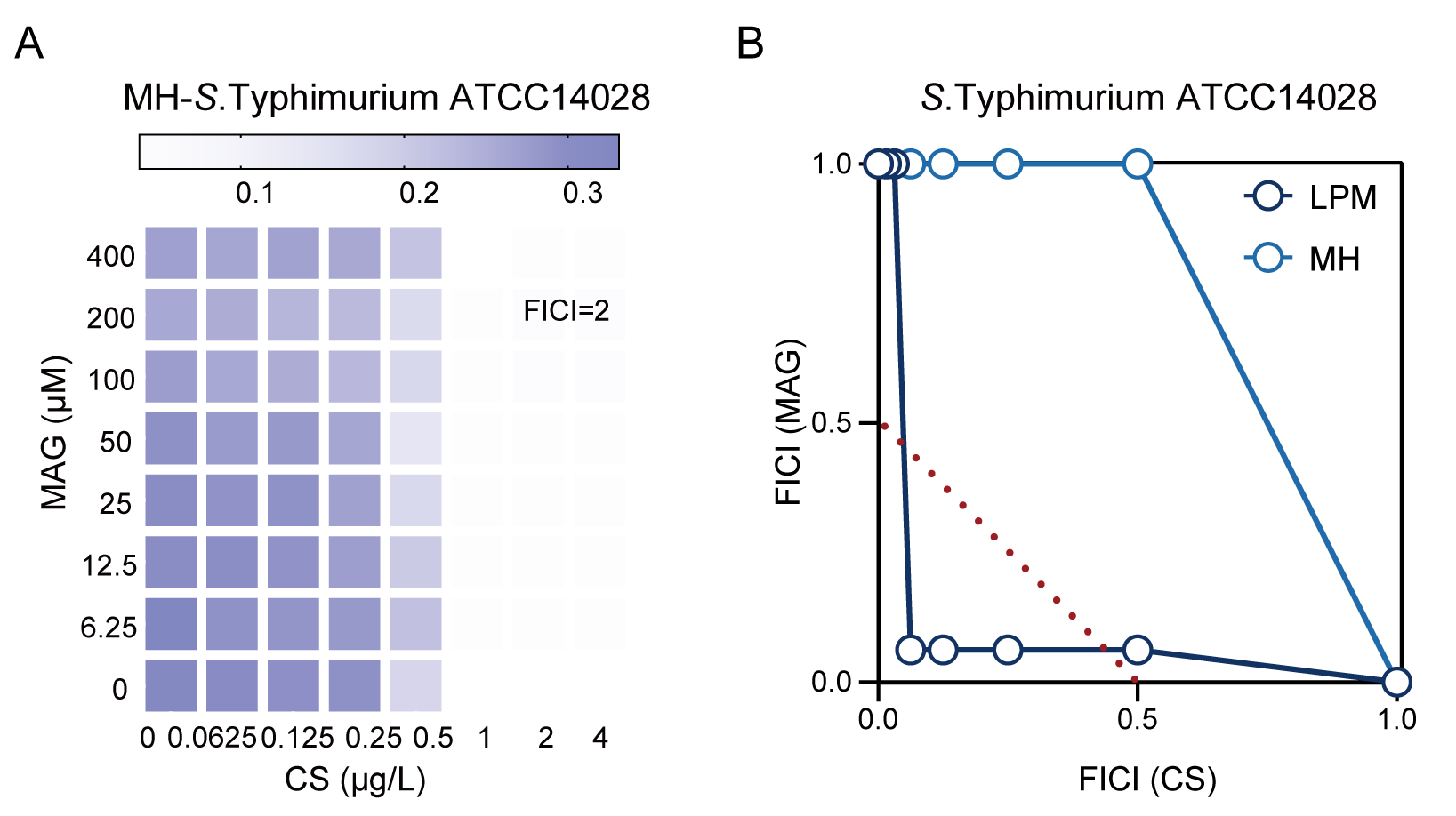

Supplement: S4 Fig — Checkerboard illustration (A); Isobologram analysis (B). (TIF) [file ppat.1013843.s004.tif]

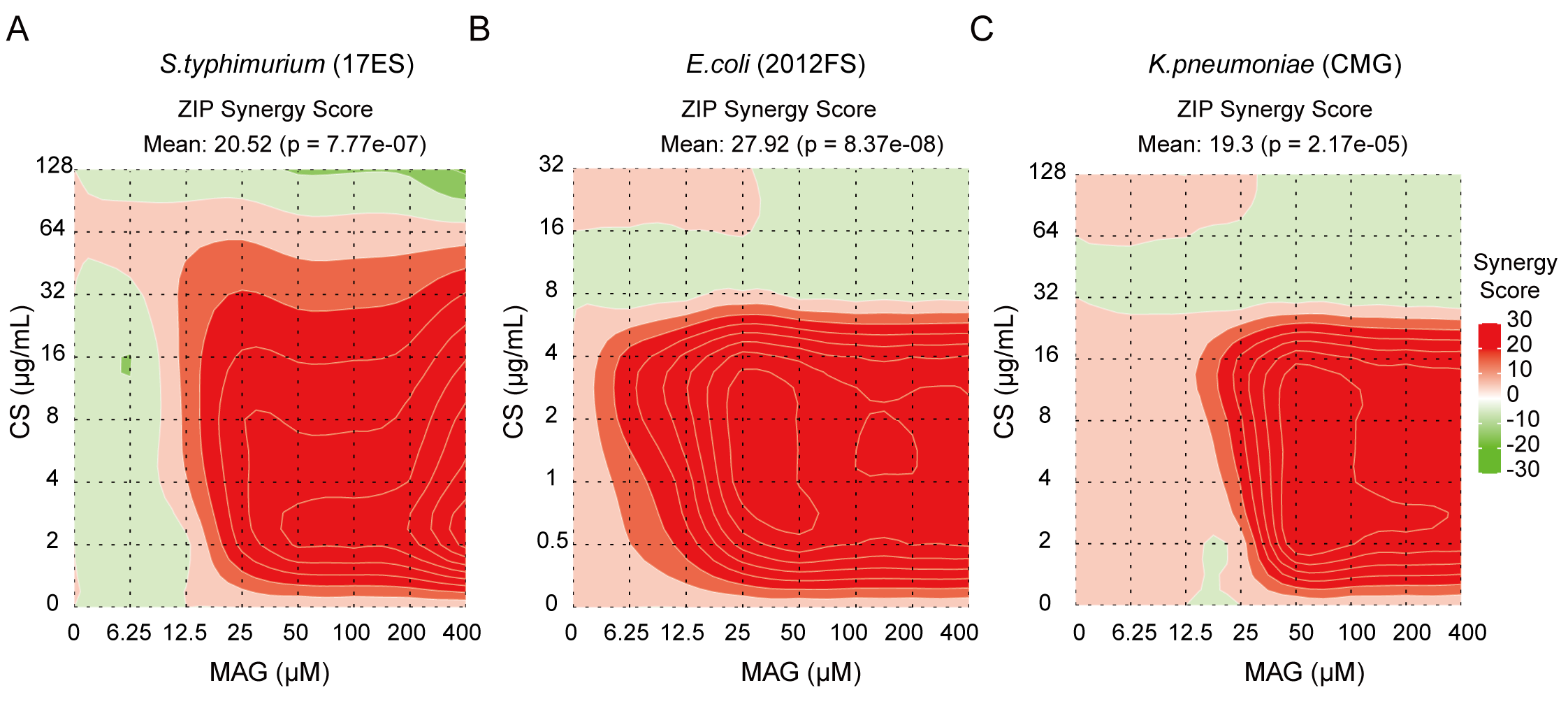

Supplement: S5 Fig — S. Typhimurium (17ES) (A), E. coli (2012FS) (B), K. pneumoniae (CMG) (C). (TIF) [file ppat.1013843.s005.tif]

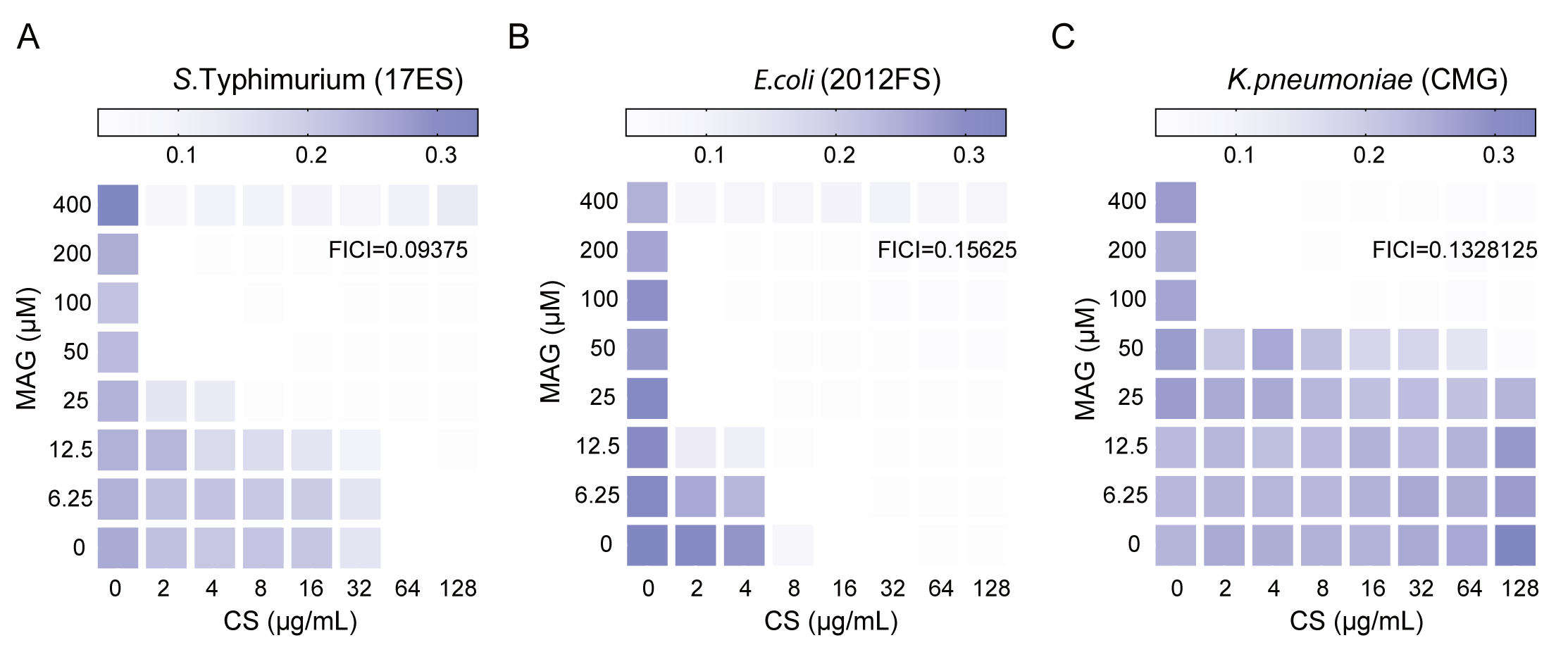

Supplement: S6 Fig — S. Typhimurium (17ES) (A), E. coli (2012FS) (B), K. pneumoniae (CMG) (C). (TIF) [file ppat.1013843.s006.tif]

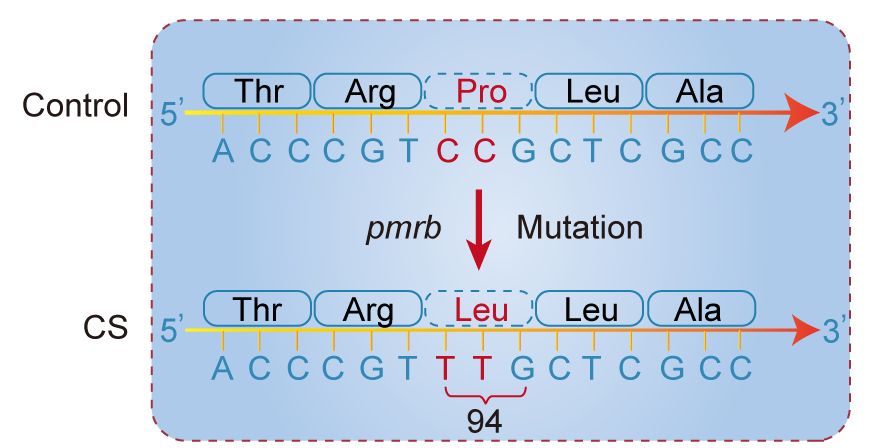

Supplement: S7 Fig — (TIF) [file ppat.1013843.s007.tif]

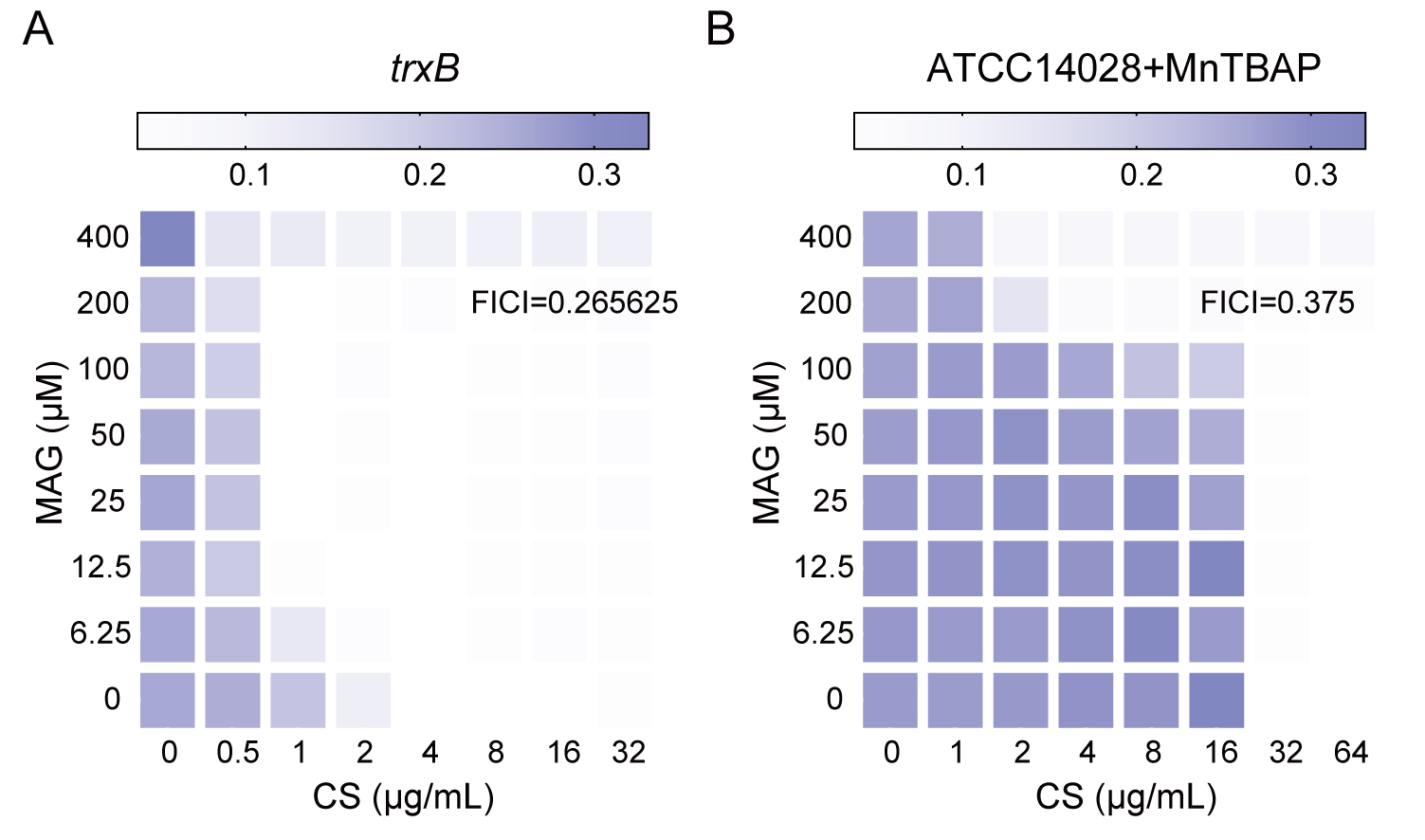

Supplement: S8 Fig — trxB deficiency reduced yet not abolished synergistic interaction between MAG and CS (A); Exogenous addition of ROS scavenger MnTBAP reduced but not diminished the synergy between CS and MAG (B). (TIF) [file ppat.1013843.s008.tif]

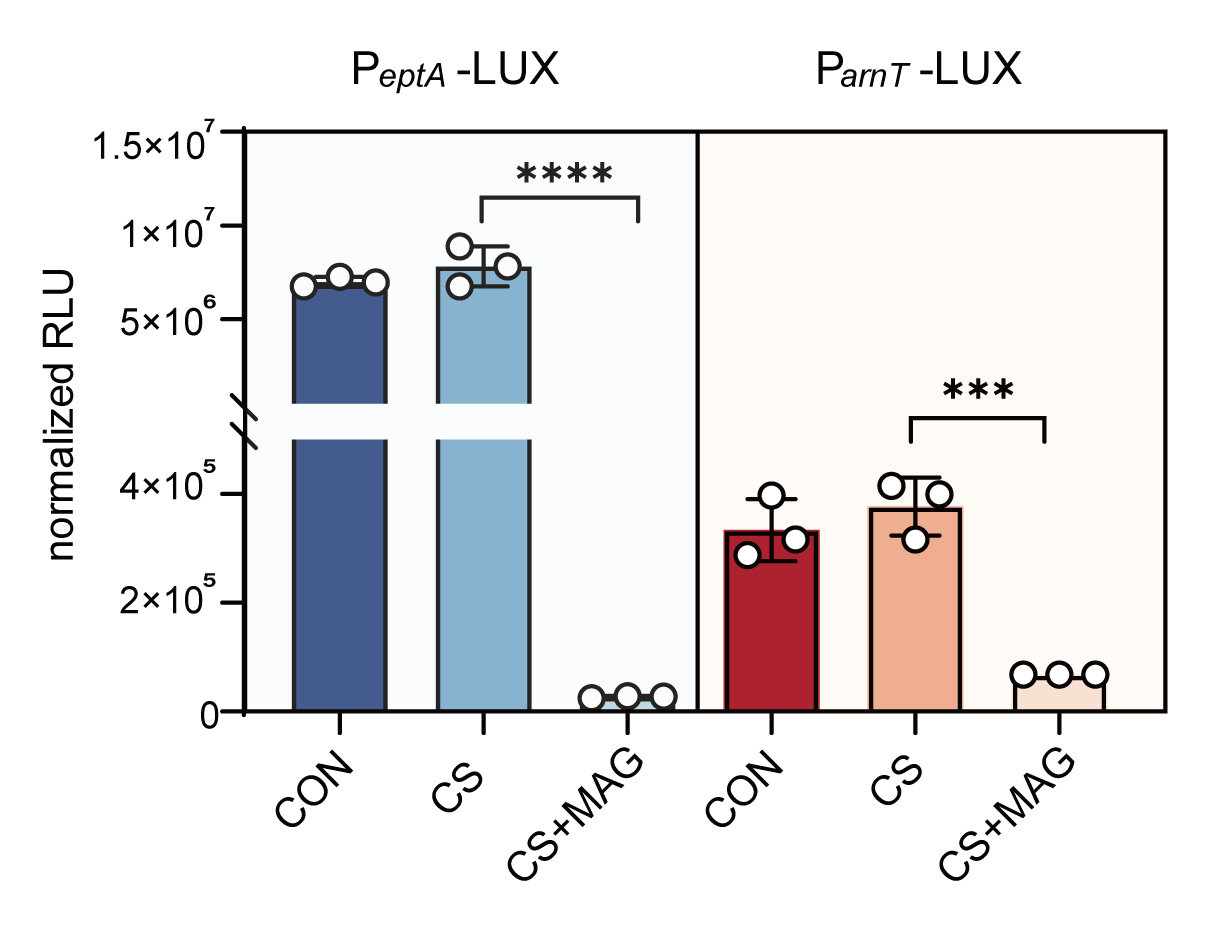

Supplement: S9 Fig — (TIF) [file ppat.1013843.s009.tif]

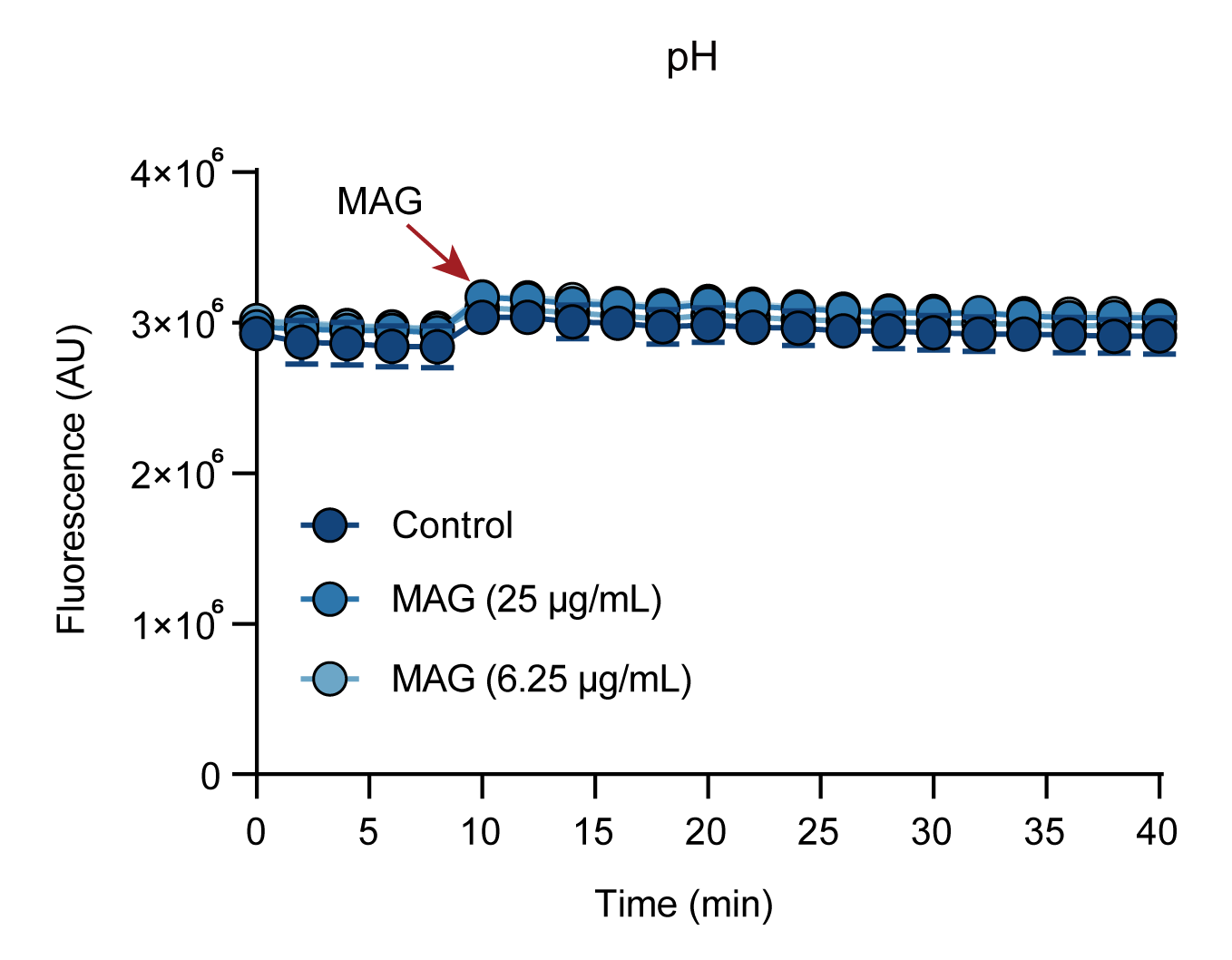

Supplement: S10 Fig — (TIF) [file ppat.1013843.s010.tif]

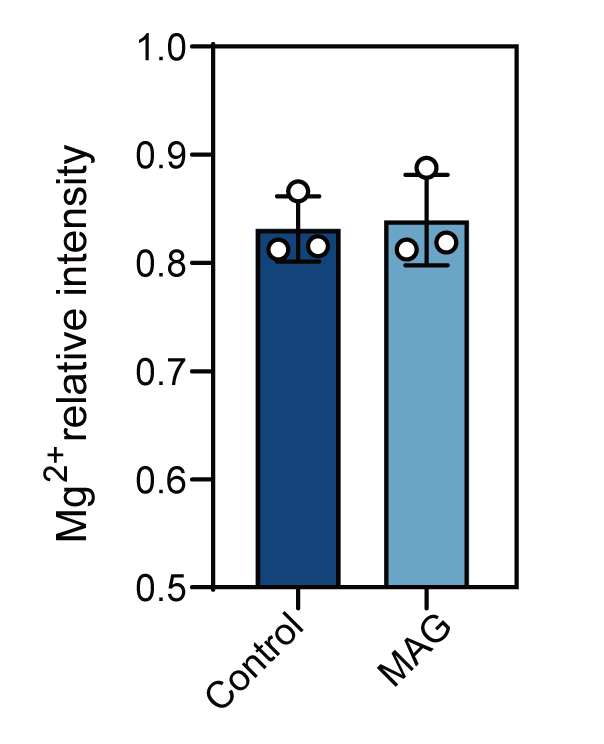

Supplement: S11 Fig — (TIF) [file ppat.1013843.s011.tif]

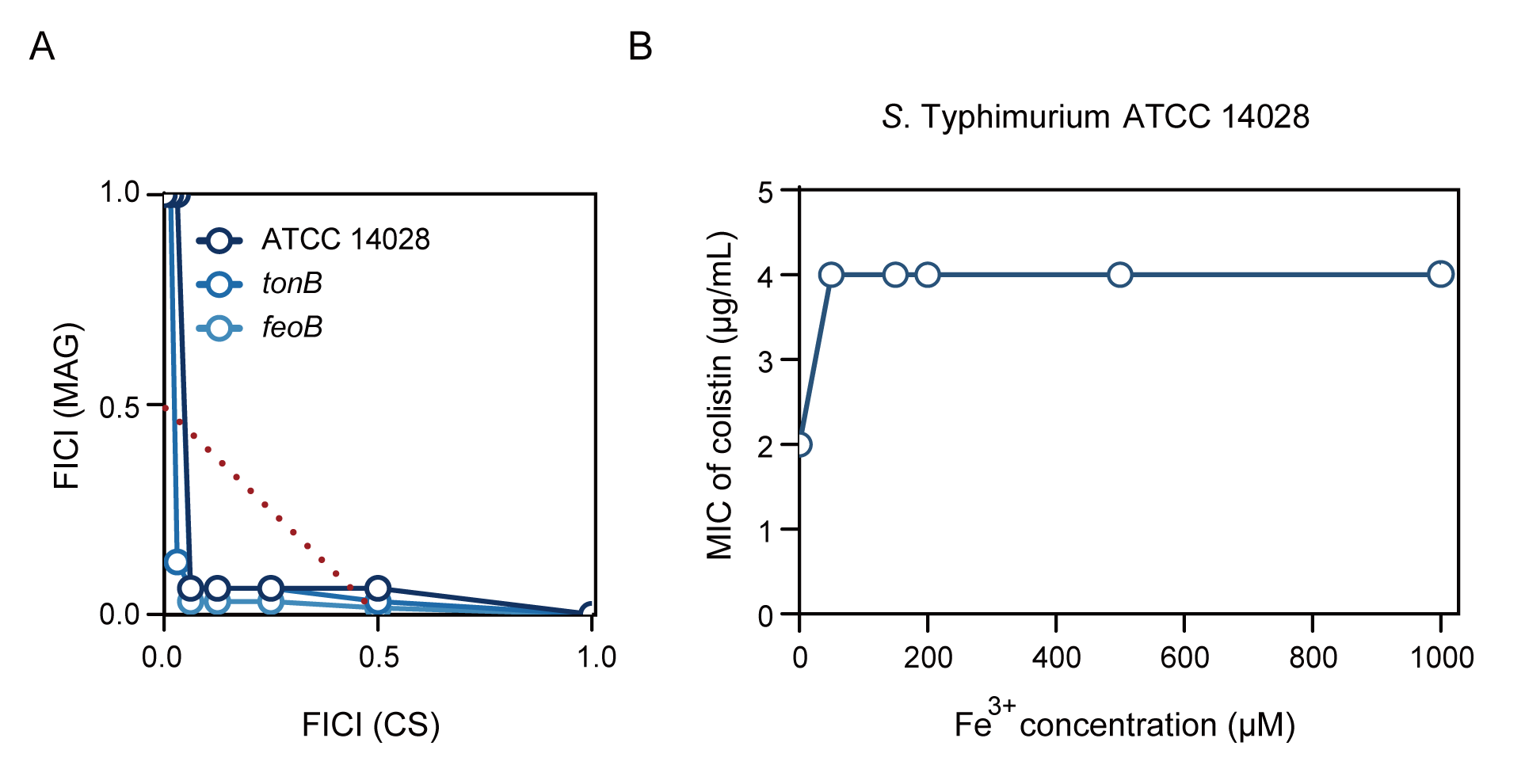

Supplement: S12 Fig — Isobolograms of the CS and MAG combination against tonB-/feoB-deficient mutants (A), The introduction of exogenous ferric iron did not abolish the synergistic effect between CS and MAG (B). (TIF) [file ppat.1013843.s012.tif]

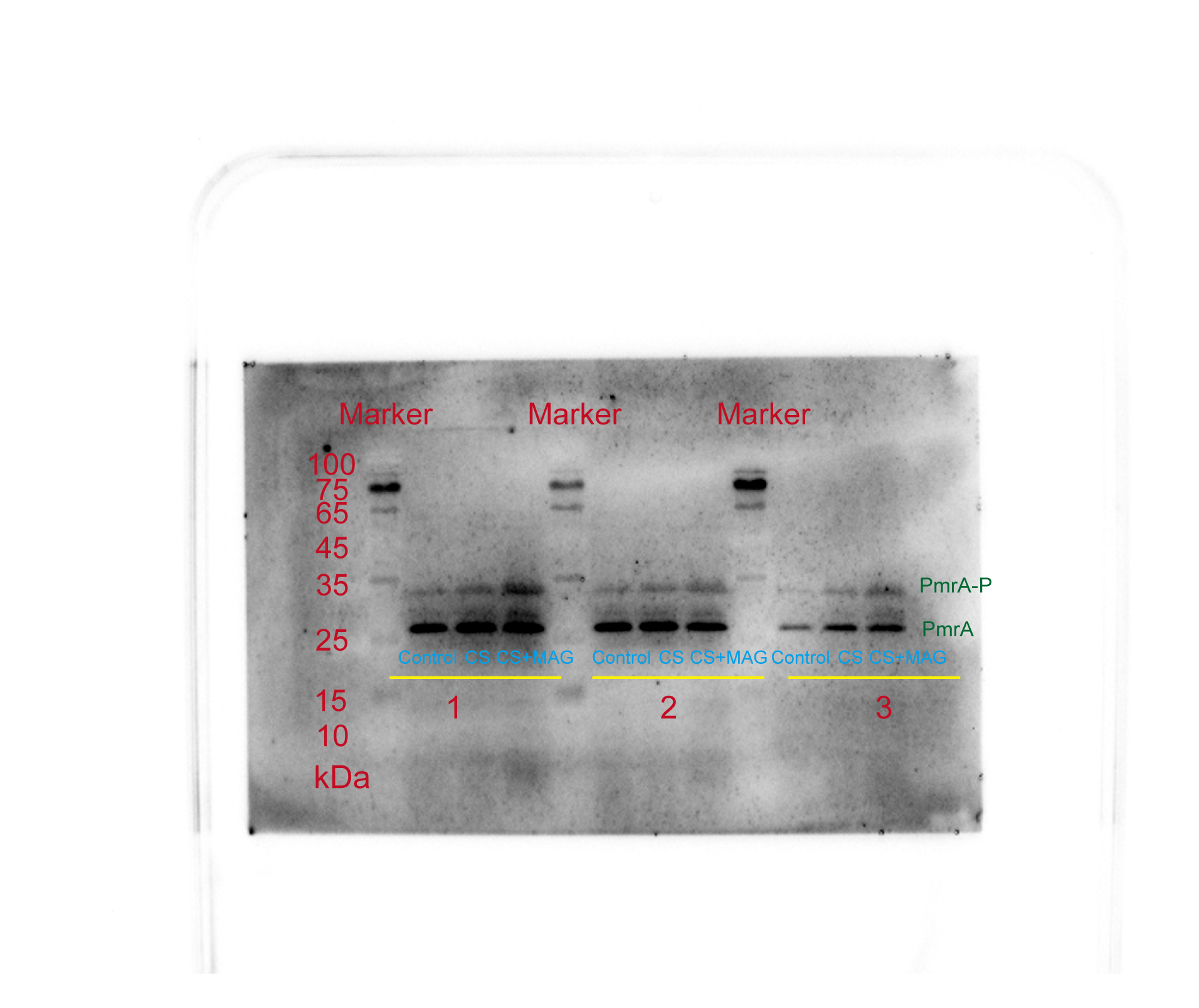

Supplement: S13 Fig — (TIF) [file ppat.1013843.s013.tif]

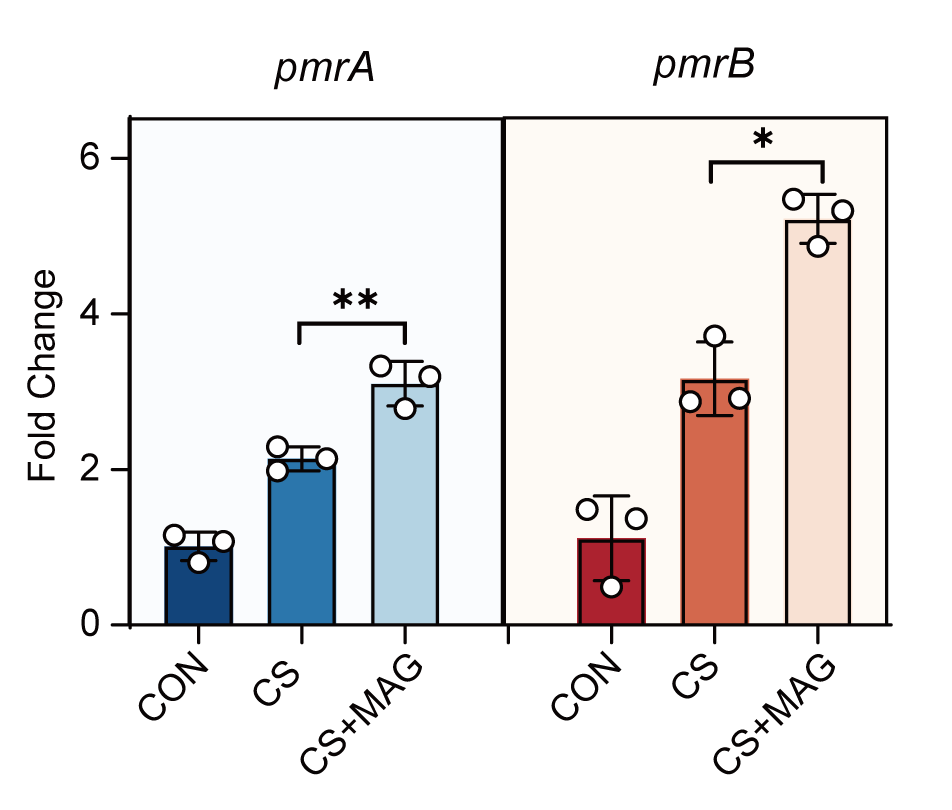

Supplement: S14 Fig — (TIF) [file ppat.1013843.s014.tif]

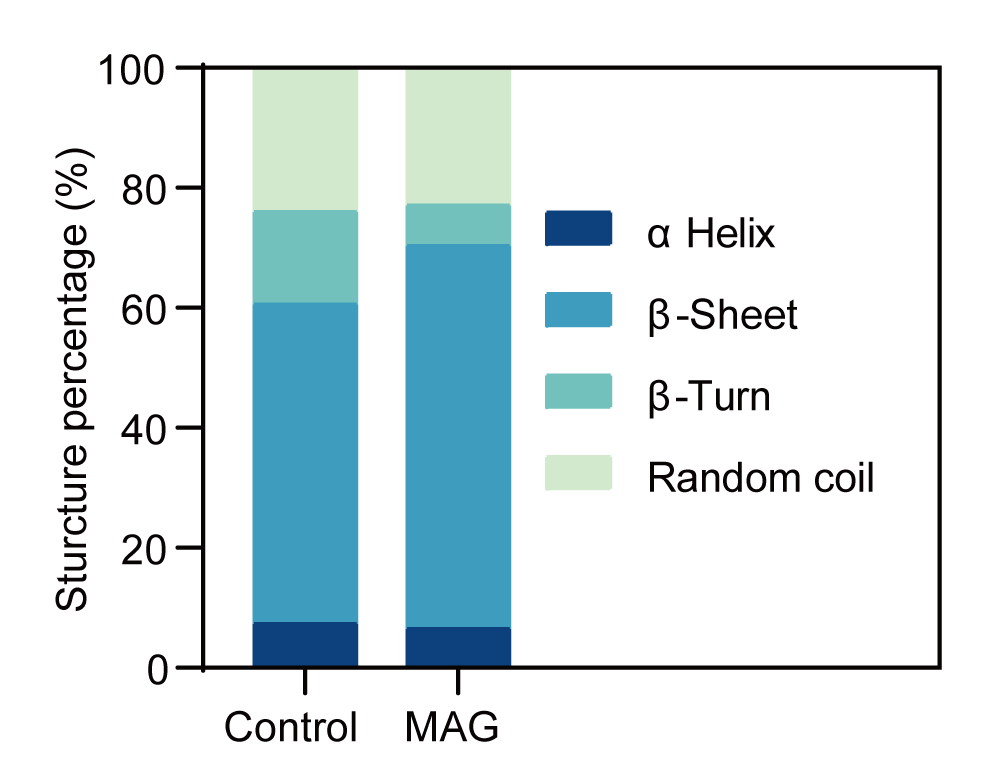

Supplement: S15 Fig — (TIF) [file ppat.1013843.s015.tif]

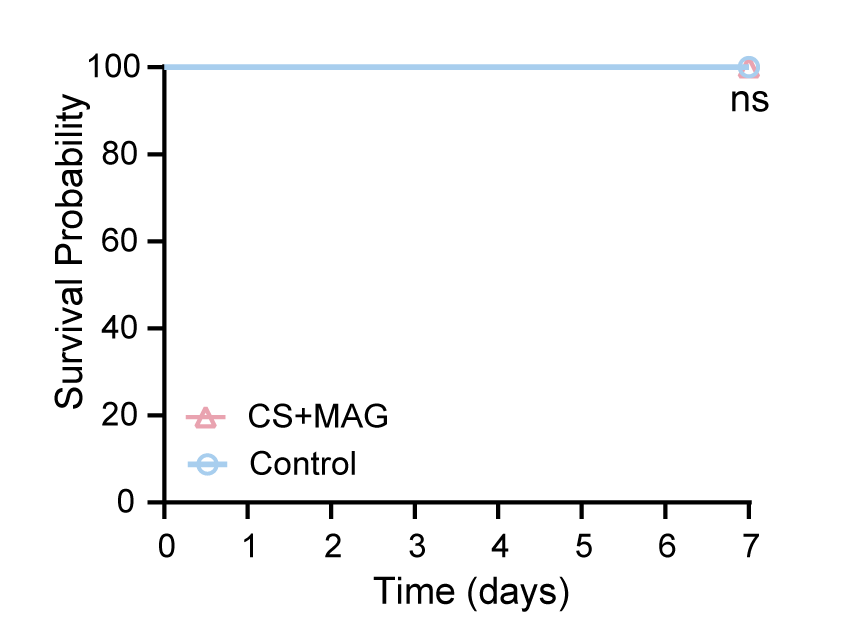

Supplement: S16 Fig — (TIF) [file ppat.1013843.s016.tif]
