# Supplementary material for: A lignan compound regulates LPS modifications via PmrA/B signaling cascades to potentiate colistin efficacy in vivo
Source: PLoS Pathog. 2025 Dec 29;21(12):e1013843. doi: 10.1371/journal.ppat.1013843 (PMC12774351; doi:10.1371/journal.ppat.1013843)
Supplement: S1 Table — (DOCX) [file ppat.1013843.s017.docx]

**S1 Table. Oligonucleotides primers used in the current study.**

| **Primers** | **Sequence (5’-3’)** |
| --- | --- |
| Q-*pmrA*-F | ACAGTCTGATGGTGCTGG |
| Q-*pmrA*-R | GTCAGATTGCCAACCGTC |
| Q-*pmrB*-F | GACCAGATGATGGATAGCG |
| Q-*pmrB*-R | GCAACAGCATACGCAGT |
| P*_arnT_*-F | AATCTGCTCTGATGCCGCATAGGATCGCACCGCTCGG |
| P*_arnT_*-R | TGAATGAAATTTTTTTAGTCATCATTTTTCCTTCAGCCATTGAAAG |
| P*_eptA_*-F | GCATAGTTAAGCCAGCCCCGAGTGGCTGGGTTGCTT |
| P*_eptA_*-R | GCCGTTAATAATGAATGAAATTTTTTTAGTCATGTTGATGCGTCCATCGATTC |
| Q-*eptA*-F | TCAACGGCTGGCGAATT |
| Q-*eptA*-R | AAGAGGTGGTATGCGG |
| Q-*arnT*-F | AAGCCGATTTCTGGCA |
| Q-*arnT*-R | AGAGAAGCGGCATGATC |
| *pmrA*-HA-F | TTGGGCTAGCAGGAGGAATTATGAAGATACTGATTGTTGAAGACG |
| *pmrA*-HA-R | GGCAATTCCGACGTCGAATTTTAAGCGTAATCTGGAACATCGTATGGGTAGCTTTCCTCAGTGGCAACC |
| Delete-*pmrB*-F | GGTTCGCGGGTTTGGCTACATGCTGGTTGCCACTGAGGAAAGCTAAGTGAATTGCAGCATTACACGTCTTGAG |
| Delete-*pmrB*-R | TGCTGATTGTCAGCAGTTTTATCTATGTGTGGGTCACGACGTATTAAACGGGCTGACATGGGAATTAGCCA |
| Delete-*pmrA*-F | GCCGCAGATGATATTCTGCAACCGTGCAGGAGACTAAGCGAATGATGCAGCATTACACGTCTTGAG |
| Delete-*pmrA*-R | CTGGCGAAGGGTCATCGCTCTTCGCTGAAAACGCATCAGGCTCACGCTGACATGGGAATTAGCCA |
| Delete-*eptA*-F | TTAACCCCTGTAATAATAGCGTGTCGTCTTCAACAATCAGTATCTGCAGCATTACACGTCTTGAG |
| Delete-*eptA*-R | TTACTTTGTCACGATTAGCGTCACCGAATCGATGGACGCATCAACGCTGACATGGGAATTAGCCA |
| Delete-*arnT*-F | TGGCCGTGAAGGTTGGCTGGGGTGTCAACAGGCAGCGAGCGCCTCGCAGCATTACACGTCTTGAG |
| Delete-*arnT*-R | GACCGCCAACGCTGAGCAGACTGGCAAGCACCAGAACGACGCCGAGCTGACATGGGAATTAGCCA |
| Delete-*trxB*-F | TGCTCATTGTCTGCCAACAACTATGGGGATCTCCAAGCTTGCATGCAGATTGC |
| Delete-*trxB*-R | GCCTTTTTTACTTTTGTTACTGATTTGTAAAAAGGCTGACATGGGAATTAGCC |
| pKD46-F | CCCGTGACAGGTCATTCAGA |
| pKD46-R | GGCACTTTTCGGGGAAATGT |
| pBAD24-F | CACGGCAGAAAAGTCCACATTG |
| pBAD24-R | GCCGAATAGCCTCTCCAC |
| pET-28a-F | GCGGATAACAATTCCCCTCTAG |
| pET-28a-R | CCAATCCGGATATAGTTCCTCC |
| *pmrA*-his6-F | GTGGACAGCAAATGGGTCGCGGATCCCATCATCACCATCACCACATGAAGATACTGATTGTTGAAGACGAC |
| *pmrA*-his6-R | TGTCGACGGAGCTCGAATTCGGATCTTAGTGGTGATGGTGATGATGGCTTTCCTCAGTGGCAACCA |
